# Diverse dietary practices across the Early Bronze Age ‘Kura-Araxes culture’ in the South Caucasus

**DOI:** 10.1371/journal.pone.0278345

**Published:** 2022-12-21

**Authors:** Nyree Manoukian, Helen L. Whelton, Julie Dunne, Ruben Badalyan, Adam T. Smith, Hakob Simonyan, Mitchell S. Rothman, Arsen Bobokhyan, Roman Hovsepyan, Pavel Avetisyan, Richard P. Evershed, A. Mark Pollard

**Affiliations:** 1 Research Laboratory for Archaeology and the History of Art, University of Oxford, Oxford, United Kingdom; 2 Organic Geochemistry Unit, School of Chemistry, University of Bristol, Bristol, United Kingdom; 3 Institute of Archaeology and Ethnography, National Academy of Sciences, Yerevan, Armenia; 4 Department of Anthropology, Cornell University, Ithaca, New York, United States of America; 5 Scientific Research Center of the Historical and Cultural Heritage, Ministry of Culture, Yerevan, Armenia; 6 Department of Anthropology, Widener University, Chester, Pennsylvania, United States of America; University of California Santa Cruz, UNITED STATES

## Abstract

The Kura-Araxes (KA) cultural phenomenon (dated to the Early Bronze Age, c. 3500/3350-2500 BCE) is primarily characterised by the emergence of a homogeneous pottery style and a uniform ‘material culture package’ in settlements across the South Caucasus, as well as territories extending to the Ancient Near East and the Levant. It has been argued that KA societies practised pastoralism, despite a lack of direct examination of dietary and culinary practices in this region. Here, we report the first analyses of absorbed lipid residues from KA pottery to both determine the organic products produced and consumed and to reconstruct subsistence practices. Our results provide compelling evidence for a diversified diet across KA settlements in Armenia, comprising a mixed economy of meat and plant processing, aquatic fats and dairying. The preservation of diagnostic plant lipid biomarkers, notably long-chain fatty acids (C_20_ to C_28_) and *n*-alkanes (C_23_ to C_33_) has enabled the identification of the earliest processing of plants in pottery of the region. These findings suggest that KA settlements were agropastoral exploiting local resources. Results demonstrate the significance of applying biomolecular methods for examining dietary inferences in the South Caucasus region.

## 1. Introduction

**Fig 1:**
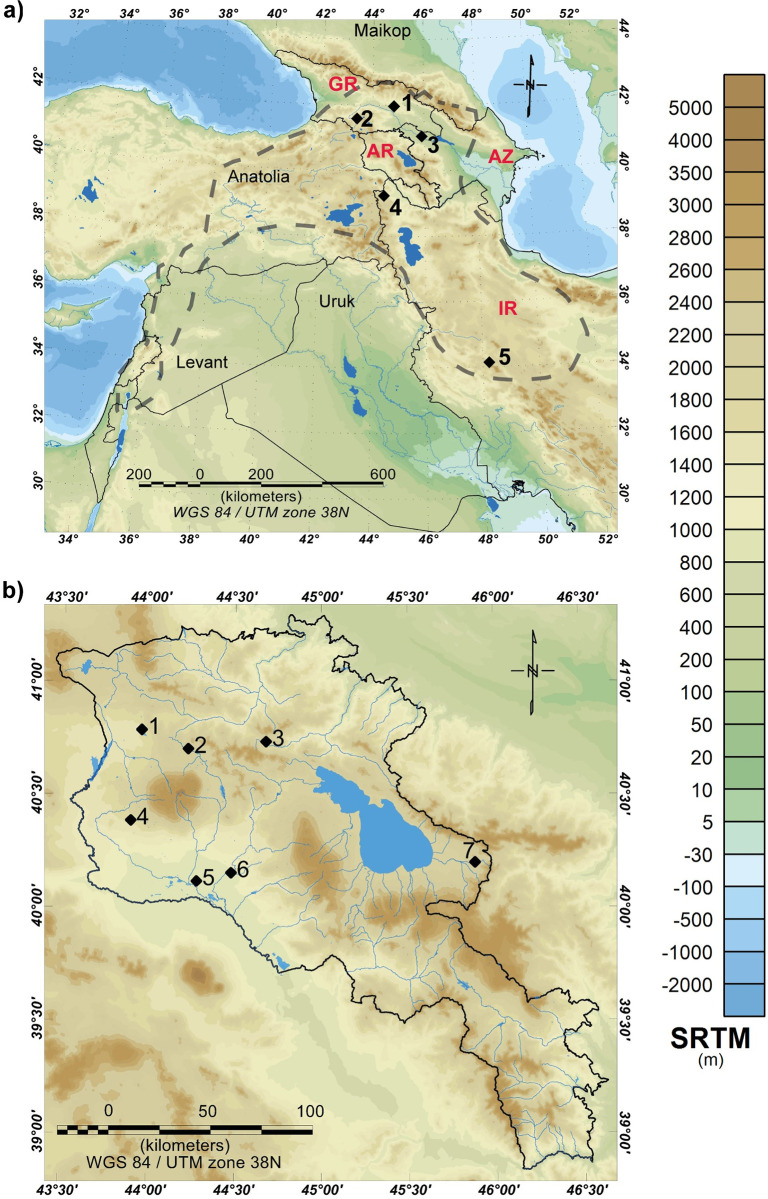
Topographic map of the Greater Caucasus (including Armenia) and the Ancient Near East. (a) Map of the Greater Caucasus and Ancient Near East displaying the extent of the Kura-Araxes cultural phenomenon in dashed lines, approximated [19] (AR is Armenia, GR is Georgia, AZ is Azerbaijan, and IR is Iran), representing sites mentioned in text (1) Natsargora and (2) Chobareti in Georgia, (3) Mentesh Tepe in Azerbaijan, (4) Köhneh Shahar and (5) Godin Tepe in Iran. (b) Map of Armenia, including rivers and one of the major freshwater lakes in the region, Lake Sevan. Sites: (1) Karnut-1, (2) Gegharot, (3) Margahovit, (4) Talin Tombs, (5) Mokhra-Blur, (6) Shengavit, and (7) Sotk-2. These settlements are distanced between 17 and 176 km, where Shengavit and Mokhra-Blur are 17 km apart. Maps produced by Lily Manoukian using Geosoft Inc. Oasis Montaj (Version 2021.2.1 [20220125–11]) (www.seequent.com); Data is public and available through Geosoft Inc.: (a) Digital Terrain Elevation Model, SRTM30 Plus v7. Data source: http://topex.ucsd.edu/WWW_html/srtm30_plus.html; (b) Digital Terrain Elevation Model, SRTM 1, Shuttle Radar Topography Mission (SRTM) 1 Arc-Second Global. Data source: https://lta.cr.usgs.gov/SRTM1Arc.

The South Caucasus has long been a pivot for cultural interaction between Europe, Eurasia and the Middle East. The Kura-Araxes (KA) cultural phenomenon, dated to the 4^th^ and 3^rd^ millennium—ca. 3500/3350-2500 BCE [1–5], emerged and spread from the South Caucasus into a much larger interconnected world (Fig 1A), and was contemporary to contrasting cultures such as the Uruk phenomenon in Mesopotamia [6], and the Maikop culture to the north [7]. KA sites are recognised by the presence of shared domestic features (architecture, tools, metallurgy, hearth features called *andirons*) and burnished grey-black-buff hand-made pottery (often called *Early Transcaucasian Ware*) from various modern territories including Armenia, Georgia, Iran, Azerbaijan, Turkey, Syria and Israel [4, 8–12]. Although its cultural expansion is vast, the South Caucasus holds key Kura-Araxes settlements pertaining to this culture, often depicted as the homeland and ‘core area’ of this cultural phenomenon [4, 10, 13]. KA settlements are characterised as heterarchical (non-hierarchical) communities [4, 14, 15]. The economic structure of these communities in terms of foodways and production require further investigation [16]. Diet and cuisine are a driving force of the development of cultural and social values within societies [16–18]. As such, questions pertaining to this culture’s social organisation, overall economy, pottery production and use, and reasons for its wide distribution are still extensively discussed [4, 9, 10].

The landscape of the South Caucasus underwent ecological, environmental, and geological transformations during the Holocene (Fig 1B). In Armenia, palaeoecological records indicate a mosaic of environments, primarily forest-steppe vegetation and semi-desert shrubs species, where the north-eastern part of Armenia is dominated by forests [20–23]. The wealth of the environment likely influenced cultures residing in various topographical zones in the South Caucasus, where Early Bronze Age (EBA) inhabitants settled in both mountainous and lowland areas (Fig 1B). Recent genetic studies point to gene flow from neighbouring populations in northern Levant during the 3^rd^ millennium BCE [24]. However, in Anatolia and the Southern Caucasus, results suggest genetic continuity, with only transient gene flow [25–27]. Movement of communities and groups suggests that subsistence practices appear to be based on transhumant pastoralism [28–30] although a dearth of archaeozoological data has precluded direct isotope analysis of animal bones [31, 32]. A vast archaeological area and previously unexplored, lipid residue analysis of KA pottery can enhance our understanding of foodways, food production and the extent of dairying in this region [33–39].

Lipid residue analysis of archaeological potsherds has become an essential tool to investigate direct evidence of production, trade, subsistence, food processing and dietary inferences [34, 40]. Lipids can be radiocarbon dated [41, 42] and analysed via gas chromatography (GC), GC-mass spectrometry (GC-MS) and GC-compound-isotope-ratio MS analysis (GC-C-IRMS), to differentiate between ruminant and non-ruminant adipose fats. Furthermore, ruminant dairy fats can be distinguished from carcass fats due to biosynthetic differences between the major fatty acids [34, 40, 43–47]. A variety of commodities can be detected, such as terrestrial animal fats [45], resins and tars [48–50], beeswax [51–54], plant products [55–63], and aquatic biomarkers [64–69].

It is postulated that KA communities were agropastoral, practising (sheep/goat) livestock farming, with secondary products (i.e., milk) being more important than primary products [16, 70, 71]. This is attested by archaeozoological evidence indicating a dominance of domestic species of cattle (*Bos taurus*), sheep (*Ovis aries*) and goat (*Capra hircus*) across KA settlements (S1 Table), including neighbouring regions such as Godin Tepe in northwestern Iran [29] and EBA Anatolian sites [72], where kill-off patterns indicate a predominance of secondary products across the region [29, 73–75]. Other domestic species include pig (*Sus scrofa*), horse (*Equus* sp.), and dog (*Canis* sp.). Moreover, cereal-based agricultural practices predominate across EBA settlements in Armenia as well as the South Caucasus region [4, 71, 76, 77]. Distinguishing between cereals used for human consumption versus fodder is difficult [78]. Cultivars include barley (*Hordeum vulgare*), naked wheat (*Triticum aestivum/turgidum*), emmer (*Triticum turgidum* subsp. *dicoccon*), and einkorn (*Triticum monococcum*) [77, 79, 80]. Plants and leafy vegetables are prevalent in the South Caucasus; for instance, Rosaceae trees, grapevine (*Vitis vinifera*), and wild forms of fig tree (*Ficus carica*) [79, 81]. Nutlets of *Lithosperumum arvense* were found at the site of Mokhra-Blur [1], and *Rubus* sp. and *Rosa* sp. (rose hip) are frequent remains of arboreal plants at Sotk-2, Gegharot, and Shengavit [71] in Armenia (S1 Table). Comparative archaeozoological and archaeobotanical data alongside lipid residue results provide a valuable lens into the socio-economic lifestyle of prehistoric populations attested in various studies [40].

Scholars claim that the KA is built on a distinct cultural identity, comprising a uniform ‘material culture package’ [1, 4, 10]. However, the extent of agricultural consumption, ruminant, non-ruminant meat, and dairying in the context of KA communities, requires further examination [16]. Previously unexplored, in terms of Kura-Araxes subsistence practices, we examine the direct evidence of absorbed residues in EBA KA vessels, to investigate the extent of dairying across settlements, the processing of other commodities and to compare diet and subsistence across seven KA settlements in Armenia (Fig 1B and S1 Table). These results hold implications for understanding the socio-economic structure of Kura-Araxes communities, in terms of reconstructing subsistence practices, foodways and identity [17, 18, 82].

## 2. Materials and methods

### 2.1 Archaeological sample selection

The archaeological sites (*n* = 7) studied here reflect varied topographical terrains (i.e., mountainous and lowland zones in the Republic of Armenia, South Caucasus) and have been selected based on the ‘core’ KA cultural area, availability of well-contextualised potsherds spanning a wide array of pottery types and chronological data (Fig 1B and S1 Table). These settlements reflect a range of different environments, due to the nature of topography and altitude. A total of 164 potsherds were analysed from seven Kura-Araxes archaeological settlements in Armenia: Gegharot (*n* = 35), Shengavit (*n* = 48), Sotk-2, (*n* = 4), Talin Tombs (*n* = 4), Karnut-1 (*n* = 30), Margahovit (*n* = 30), and Mokhra-Blur (*n* = 13) (Fig 1B). The contextual information for potsherd samples is provided in S1 and S2 Tables. Permission to perform scientific analyses on potsherd samples from settlements in Armenia was granted by researchers at (1) the Institute of Archaeology and Ethnography, National Academy of Sciences, and (2) Scientific Research Center of the Historical and Cultural Heritage, Ministry of Culture in Yerevan. Potsherd samples for this study were obtained and studied in Summer 2014 for Shengavit; Summer 2017 for Shengavit, Gegharot, Margahovit, Sotk-2, Mokhra-Blur, and Talin Tombs; and Summer 2019 for additional potsherds from Gegharot and Karnut-1. For accessibility and research purposes, any individual and/or group can seek access to the materials from these institutions by contacting co-authors who are directors of the archaeological sites mentioned above and affiliated with the institutions. Additional information regarding the ethical, cultural, and scientific considerations specific to inclusivity in global research is included in the Supporting Information (S1 Checklist). The described study complied with all relevant regulations.

While the sample sizes for Sotk-2 and Talin Tombs are low, these sites will be discussed in combination with all sites to cover overall trends. Rim and upper body sherds were selected for analysis, where possible, due to higher concentration of lipids preserved, specifically from boiling foodstuffs [48]. Potsherds selected are primarily classified as utility vessels, burnished and unburnished; cooking pots were recognised through the presence of sooting clouds indicating vessel heating over a fire [83]. Further assessment on vessel design and shape was limited due to the selection of fragmentary potsherds instead of whole vessels. Well-contextualised potsherds correspond to the (EBA) KA occupational excavated layers at all settlements; however, direct radiocarbon dates for potsherds in this region are still lacking and require further research [41, 42].

### 2.2 Lipid extraction

Lipid analysis and interpretations were performed using established protocols, discussed in detail in various publications [58, 78, 84]. All solvents used were HPLC grade (Rathburn) and the reagents were analytical grade (typically >98% of purity). All glassware was washed with Decon 90 (Deacon Laboratories), acetone and dichloromethane (DCM), and further sterilized in the oven at 450°C for 4 h. The syringes used during the extraction were solvent-rinsed (*n*-hexane, x10 and ethyl acetate, x10) before and after their use, and all the other tools were solvent-sonicated (15min in DCM). For each lipid extraction batch, analytical blanks were prepared in order to detect potential contamination from solvents or reagents.

Briefly, ca. 2 g of potsherd were cleaned with a modelling drill to remove exogenous lipids, and further ground to homogenous powder, and transferred into furnaced culture tubes. An internal standard was added to enable quantification of the lipid extract (*n*-tetratriacontane, typically 40 μg), prior to methanolic acid extraction. The lipids were then esterified and/or transesterified using 5 mL of 2% sulfuric acid/methanol solution (δ^13^C value measured) and heated for 1 h at 70°C mixing every 10 min. The supernatant was removed to a clean test-tube and 2 mL of (DCM) extracted double-distilled water added. The remaining potsherd was washed with 5 mL of *n*-hexane and transferred to test-tubes before centrifuging (2500 rpm, 10 min). The *n*-hexane supernatant was then transferred to the sulfuric acid-methanol solution and whirlimixed to extract the lipids before being transferred to a vial, and a further 3 × 3 mL of *n*-hexane was added to the H_2_SO_4_-methanol solution. The extracts were combined, and the solvent was removed under a gentle stream of nitrogen in a heating block at 40°C. An aliquot of the extract was treated with *N*,*O*-bis(trimethylsilyl)trifluoroacetamide (BSTFA) containing 1% *v/v* trimethylchlorosilane (Sigma Aldrich) prior to analysis via GC-FID, GC-MS and GC-C-IRMS.

### 2.3 Instrumental analysis: GC-MS and GC-C-IRMS

Instruments and instrument conditions for GC-MS followed protocols outlined in various publications [78]. Analyses of acid extracted FAMEs TLEs were performed using an Agilent 7890A gas chromatograph. The FID used to monitor column effluent was set to 300°C. Trimethylsilylated FAMEs were introduced to the system via on-column injection (1.0 μL). Data was acquired using HP Chemstation software (Rev. C.01.07(27) Agilent Technologies) and eluted peaks were identified by comparison of retention times with those of an external standard (FAME), quantification was calculated using a known amount of internal standard introduced during sample preparation.

GC-MS analyses of trimethylsilylated FAME aliquots were performed using ThermoScientific Trace 1300 gas chromatograph coupled to an ISQ single quadrupole mass spectrometer. Samples were introduced to an injector set to splitless mode. The GC temperature programme was set to hold at 50°C for 1 min, followed by a gradient increase to 300°C at 10°C min, once at 300°C the oven was run isothermally for 10 min. The MS was operated in electron ionisation (EI) mode operating at 70 eV, with a GC transfer line temperature of 300°C and a source temperature of 300°C. The emission current was set to 150 μA and the MS was set to acquire in the range of *m/z* 50–650 at 2 scans s^-1^ in full scan mode.

For the detection of *ω*−(*o-*alkylphenyl)alkanoic acids (APAAs) and isoprenoid fatty acids, TLEs were injected onto a 60 m × 0.32 mm fused silica capillary column coasted with a VF-23ms stationary phase (50% cyanopropyl-methylpolysiloxane, Varian, Factor Four 0.15 μm). The GC temperature programme was set to hold at 50°C for 2 min, followed by a gradient to 100°C at 10°C min^-1^ and then to 240°C at 4°C min^-1^ before a final isothermal at 240°C for 15 min. Helium was used as the carrier gas and maintained at a constant flow of 2 mL min^-1^. The MS was operated in electron ionisation (EI) mode operating at 70 eV, with a GC transfer line temperature of 250°C and a source temperature of 200°C, the emission current was set to 150 μA. The MS was set to operate in selected ion monitoring (SIM) mode, scanning for the molecular ions (M^+•^) for APAAs of carbon chain lengths C_16_-C_22_ at *m/z* 262, 290, 318 and 346 and the fragment ion of the base peak *m/z* 105 [65], and IFAs (the fragmentation of 4,8,12-trimethyltridecanoic acid was identifiable with ions *m/z* 87, 213, 270; pristanic with ions *m/z* 88, 101, 312; and phytanic with ions *m/z* 101, 171, 326). Data acquisition and processing were carried out using XCalibur software, version 3.0. Compounds were identified by comparison with the NIST mass spectra library (version 2.0) or with reference to external sources such as The Lipid Library (www.lipidlibrary.aocs.org), for the identification of APAAs mass chromatograms were compared to an archaeological standard known to contain C_16_, C_18_, C_20_ and C_22_ APAAs.

Compound specific carbon stable isotope analyses were performed using an Agilent Industries 7890A gas chromatograph coupled to an IsoPrime 100 mass spectrometer. Samples were introduced via a split/splitless injector in splitless mode onto a 50 m × 0.32 mm fused silica capillary column coated with a HP-1 stationary phase (100% dimethylpolysiloxane, Agilent, 0.17μm). The GC oven temperature programme was set to hold at 40°C for 2 min, followed by a gradient increase to 300°C at 10°C min^˗1^, the oven was then run isothermally for 10 min. Helium was used as a carrier gas and maintained at a constant flow of 2 mL min^˗1^. The combustion reactor consisted of a quartz tube filled with copper oxide pellets which was maintained at a temperature of 850°C. Instrument accuracy was determined using an external FAME standard mixture (C_11_, C_13_, C_16_, C_18_, C_21_, and C_23_) of known isotopic composition (determined using a two-point calibration using IAEA-CH-7 (δ^13^C value = -32.2 ± 0.05‰) and FIRMS phenacetin (δ^13^C value = -26.7 ± 0.2 ‰). Samples were run in duplicate and an average recorded (difference between duplicates < 0.5 ‰). The δ^13^C values are the ratios ^13^C/^12^C and expressed relative to the Vienna Pee Dee Belemnite, calibrated against a CO_2_ reference gas of known isotopic composition. Instrument error was ±0.3‰. Data processing was carried out using Ion Vantage software (version 1.5.6.0, IsoPrime Elementar).

## 3. Results

### 3.1 Lipid preservation

A suite of different lipid classes was detected within the pottery vessels, the most abundant of which were degraded animal fats in the form of saturated fatty acids. Other lipid classes detected comprise aliphatic lipids including *n*-alkanes and *n*-alkanols. A summary of lipids detected is given in S2 Table. Analyses yielded interpretable lipid concentrations (>5 μg g^-1^) from 91.5% of 164 sampled potsherds (S1 Fig). Lipid preservation was remarkably high for all the sites examined, specifically at Mokhra-Blur (100%, *n* = 13), Talin Tombs (100%, *n* = 4) and Sotk-2 (100%, *n* = 4) where all sherds sampled yielded lipids, and still quite high at the sites of Margahovit (73%, *n* = 22), Karnut-1 (60%, *n* = 18), Shengavit (66%, *n* = 32), and Gegharot (61%, *n* = 22) (Table 1). Of these, the average concentration of extracted lipids was 244.3 μg g^-1^, the highest being 3384.9 μg g^-1^. A total of 115 (70.2%) sherds across all sites contained sufficient lipids for compound-specific isotopic analysis.

**Table 1:** Summary of occurrence of lipid classes detected in pottery vessels at each site.

Site	Period	% lipid recovery	Av. Lipid concentration (μg g^-1^)	Animal resources			Aquatic resources	Plant resources
				NRA	RA	RD		Aliphatic lipids
**Shengavit**	KA I-II	94	317.4	3	25	4	3	10
**Mokhra-Blur**	KA I-II	100	291.1	0	9	4	1	5
**Margahovit**	KA II	90	45.0	1	14	7	4	9
**Gegharot**	KA I-II	77	305.2	0	10	12	1	2
**Karnut-1**	KA II	100	95.3	10	4	4	0	6
**Talin Tombs**	KA I	100	262.1	0	1	3	0	0
**Sotk-2**	KA I-II	100	116.9	0	2	2	0	2

Average lipid concentration of sherds containing a significant lipid concentration (>5 μg g^-1^ of potsherd). NRA = non-ruminant adipose, RA = ruminant adipose, RD = ruminant dairy. Aquatic resources include the co-occurrence of C_18_, C_20_, and C_22_ APAAs and/or isoprenoid fatty acids (TMTD, phytanic and pristanic acids). KA phase I extends from 3600/3500-2900 BCE, while KA phase II ranges from 2900-2600/2500 BCE [2].

### 3.2 Terrestrial resources

**Fig 2:**
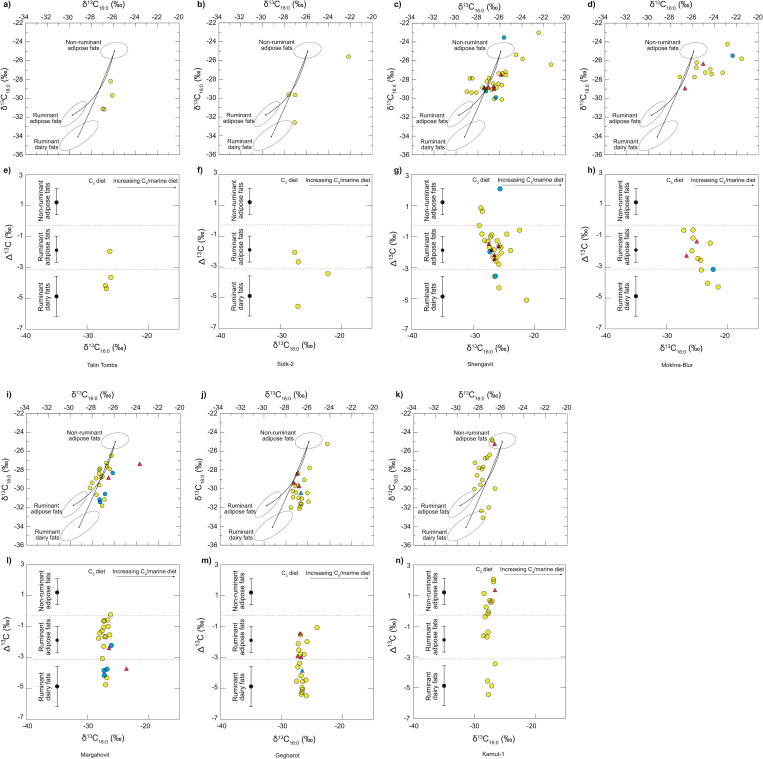
Combined scatter plot showing δ^13^C values of the major fatty acid components (*n*-C_16:0_ and *n*-C_18:0_) prepared from lipid extracts from examined sites. Shengavit (c, j), Gegharot (f, m), Karnut-1 (g, n), Margahovit (e, l), Sotk-2 (b, i), Mokhra-Blur (d, k) and Talin Tombs (a, h). Each data point corresponds to an individual vessel. Residues with evidence of plant processing, see S3 Table for labelled data points. Plots (**a-g**): The values of reference fats are represented by confidence ellipses (±1σ) for animals raised on a strict C_3_ diet [46]. The three annotated fields correspond to P = 0.684 confidence ellipses relating to modern fats of animals raised on a purely C_3_ diet in Britain [46]. Plots (**h-n**): The difference in the δ^13^C values of the *n*-C_18:0_ and *n*-C_16:0_ fatty acids (Δ^13^C = δ^13^C_18:0_ - δ^13^C_16:0_) obtained for the *n*-C_16:0_ and *n*-C_18:0_ fatty acids prepared from lipid extracts from all sites. Ranges depicted represent the mean ± 1 s.d. of Δ^13^C values for a global database published elsewhere comprising modern reference animal fats from various geographical settings that include: Africa [86], UK [44], Kazakhstan [95], Switzerland [96], and the Middle East [97]. Triangle symbols represent TLEs with evidence of plant processing with CPI values >5; blue symbols represent TLEs with evidence of aquatic components (APAAs and IFAs). Analytical precision is ±0.3‰.

Characterisation of degraded fats was achieved through stable carbon isotopic values (δ^13^C) of the major fatty acids (*n*-C_16:0_ and *n*-C_18:0_) (Fig 2). Degraded ruminant adipose products were the most common class of lipid detected in Kura-Araxes potsherds, at 56% (Table 1). The δ^13^C values obtained indicate the animals were raised primarily on a C_3_ diet ([46]; marked by the confidence ellipses). However, the δ^13^C values of the fatty acids in the TLEs (total lipid extract) from the sites of Shengavit, Sotk-2, and Mokhra-Blur exhibit an isotopic shift (Fig 2B–2D). The isotopic shift is generally indicative of environmental factors, such as aridity [85], warm climate [78, 86, 87], and animals grazing on salt marshes [88, 89], which can broaden the δ^13^C values. As a result, lipids from these sites have been classified using their Δ^13^C (= δ^13^C_18:0_ –δ^13^C_16:0_) values (Fig 2). In general, the δ^13^C values vary over a wide range within each settlement, suggesting a variety of vegetation types and microenvironments [90, 91]. At the archaeological settlement of Shengavit, δ^13^C_16:0_ values of herbivore fatty acids range between -29.0 and -21.3‰. Similarly, Mokhra-Blur’s δ^13^C_16:0_ values of herbivore fatty acids range between -27.2 and -21.5‰. The enrichment of these values suggests some input of C_4_ plants in the diet of ruminants and/or non-ruminants, as exhibited in the North Caucasus paleoenvironments [92, 93]. Domesticated C_4_ crops are absent during the EBA, as millet does not appear in the archaeological record until the Middle/Late Bronze Age in South Caucasus [71, 80]. However, wild C_4_ vegetation and salt marshes are common in the region of Armenia, specifically in the Ararat Plain, where Shengavit and Mokhra-Blur settlements are located [94] (S3 Fig). Lipid extracts with less depleted δ^13^C_16:0_ and δ^13^C_18:0_ values (potsherds from Gegharot and Karnut-1) appear to have originated from an animal fat source. Due to the significantly low amount (8%) of aquatic resources detected, it is not enough to suggest the cause of enrichment; thus, animals grazing and foddering on salt marshes in this region, is likely the cause of enriched δ^13^C values causing offset from the confidence ellipses [88, 89, 94].

Dairy products, in comparison to ruminant carcass fats, were lower than expected (31%), in contrast to the various archaeozoological studies which report the importance of secondary products [70]. However, dairying seems to dominate more at Gegharot (55%), Talin Tombs (75%), and Sotk-2 (50%) (Fig 2), than at other settlements (Table 1). Pigs are represented in EBA KA sites across the South Caucasus, but in very low numbers [19, 77, 79]. Non-ruminant resources are evident at Shengavit (9%) and Karnut-1 (55%).

### 3.3 Plant exploitation and processing

**Fig 3:**
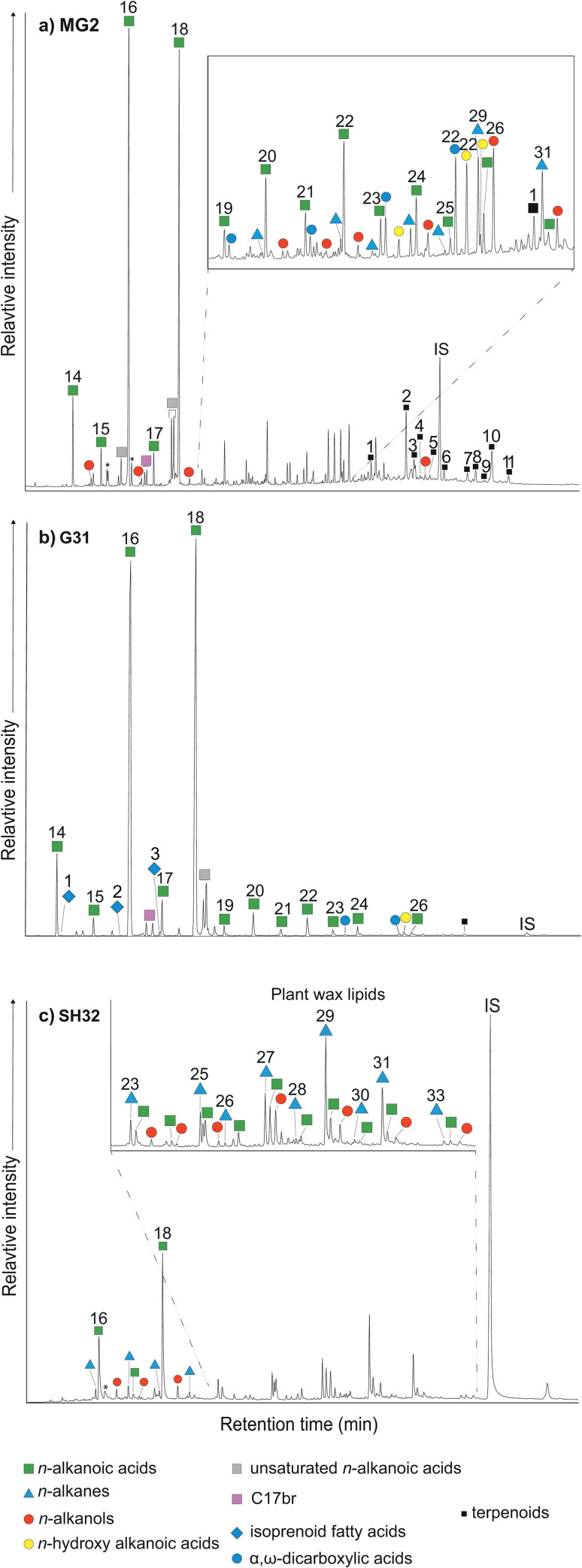
Chromatograms depicting plant lipids, terrestrial and aquatic fats. (a) Partial GC-FID chromatogram from Margahovit (MG2) depicting birch bark tar biomarkers, numbered black squares; (1) Lup-2,20(29)-diene, (2) Lup-2,20(29)-dien-28-ol (TMS ether), (3) Allobetul-2-ene, (4) Lupeone, (5) Lupeol (TMS ether), (6) unknown terpenoid, (7) 28-oxoallobetul-2-ene, (8) Betulone (TMS ether), (9) 3-oxoallobetulane, (10) Betulin (TMS ether), (11) Allobetulinol (TMS ether); (b) partial GC-MS chromatogram from Gegharot (G31) classified as a dairy residue with isoprenoid fatty acids: 4,8,12-trimethyltridecanoic acid (TMTD), pristanic and phytanic acids; (c) partial GC-MS chromatogram from Shengavit (SH32) illustrating the distribution of compounds characteristic of plant lipids with a CPI of 18.8. Plasticisers are marked with asterisks. IS, internal standard, C_34_ *n*-tetratriacontane.

Evidence for the exploitation of plants is found at all sites, except Talin Tombs (Table 1). In particular, plant aliphatic lipids are recorded at Sotk-2 (50%), Shengavit (45%), Margahovit (41%), Mokhra-Blur (38%), Karnut-1 (33%) and in low abundance at Gegharot (9%) (S2 and S3 Tables). Abundant *n*-alkanes with an odd-over-even dominance were observed in extracts lacking high abundance of animal fats, implying the processing of plants in some vessels (Fig 3C). Diagnostic long-chain fatty acids from C_20:0_-C_28:0_, high abundances of C_18:1_ unsaturated fatty acids, odd-numbered *n*-alkanes ranging from C_19:0_ to C_33:0_, even-numbered *n*-alkanols ranging from C_14:0_ to C_30:0_, *n*-hydroxy fatty acids, and short-, medium- and long-chain diacids were detected (Fig 3A and 3C). Such distributions are possibly characteristic of plant wax and/or seed oils [58, 62, 98, 99]. Overall, 29% of potsherds contained lipid profiles that can be characterised as plant. *n*-Alkane distributions have been calculated for the carbon preference index CPI [99, 100] and *P*_aq_ proxy ratio [58, 101] (S3 Table). The CPI was calculated to determine whether the *n*-alkanes derived from epicuticular plant waxes or from post-depositional contamination [102–105]. The δ^13^C values for the series of *n*-alkanes derived from terrestrial plant waxes range from -35.6 to -24.3‰. These reflect the carbon isotope values of C_3_ leaf wax lipids, which range between -39‰ and -29‰, and some C_4_ input due to salt marsh and water-stressed vegetation [103] (S3 Fig).

Triterpenoids were also identified in five extracts from Margahovit (MG2), Karnut-1 (KRT35), Gegharot (G28 and G31) and Shengavit (SH132) (Fig 3A). Identification of triterpenoids was conducted using GC-MS, as the components displayed base peaks at *m/z* 189 and molecular ions (M^+•^) at *m/z* 426, 498 and 468; comparisons with reference mass spectra identified the characteristic components of birch bark tar; lupeol, lupeol and betulin in conjunction with their degradation markers produced via oxidation and heating e.g., allobetul-2-ene, 3-oxoallobetulane, and 28-oxoallobetul-2-ene [106–108]. Diterpenoids deriving from the oxygenated pyrolysis of *Pinus spp*., dehydroabietic acid and 7-oxodehydroabietic acid, were detected in TLEs from Mokhra-Blur (77%) and Karnut-1 (30%) and these are likely to have derived during firing of the pottery [109] as forests comprising hazel, fir, and pine species were abundant in the area during the EBA [20–23].

### 3.4 Aquatic commodities

All potsherds containing an appreciable amount of lipids were screened using GC-MS in selected ion monitoring (SIM) mode for the presence of *ω*−(*o*-alkylphenyl)alkanoic acids (APAAs) by scanning for the molecular ions (M^+•^) for APAAs of carbon chain lengths C_16:0_-C_22:0_ at *m/z* 262, 290, 318 and 346 and the fragment ion of the base peak *m/z* 105. Aquatic biomarkers (APAAs C_18:0_-C_20:0_ and traces of C_22:0_, and isoprenoid fatty acids) were detected in 9 extracts (8%) (Fig 3B, Table 1 and S2 Fig). Six of the extracts (G31, SH9, MB3, MG9, MG29, and MG33), contained all three isoprenoid fatty acids (IFAs), and an absence of APAAs. IFAs derive from phytol in marine algae [65, 68]; 4,8,12-trimethyltridecanoic acid (TMTD), 2,6,10,14-tetramethylpentadecanoic acid (pristanic acid), and 3,7,11,15-tetramethylhexadecanoic acid (phytanic acid). The presence of APAAs in four lipid extracts (MB3, MG17, SH48 and SH68) suggests that the aquatic commodities were heated to high temperatures of >200°C in the pottery vessels [110, 111].

## 4. Discussion

The biomolecular analysis of lipids from potsherds from Kura-Araxes settlements across Armenia provides strong evidence for a diversified diet. These findings provide the first direct evidence of the mixing and processing of various commodities, including plants, leafy vegetables, and/or fruit, birch bark tar, offering a new outlook on the overall economic and subsistence strategies of Bronze Age people in the core area of the KA culture (Figs 2, 3, and S2 Fig). The current view suggests that KA communities are linked by a highly distinctive material cultural package, comprising similar architecture, material culture, such as tools, metallurgy, and pottery styles, despite settlements being distributed across a vast territorial region [4, 8–10]. Our study demonstrates diverse subsistence practices occur across all settlements within Armenia, South Caucasus, and there are detectable differences between and within highly contrasting environments, including mountains (Gegharot, Margahovit, Karnut-1, Talin Tombs and Sotk-2) and lowland river-based settlements (Mokhra-Blur and Shengavit). These findings from lipid residue analysis of Kura-Araxes pottery suggest that diversification in foodways was quite common in the EBA period (KA I-II; S1 Table), in contrast to neighbouring regions exhibiting a homogeneous diet, such as coeval Kura-Araxes settlements in Azerbaijan [31] and EBA settlements in Anatolia [72]. Lipid residue analysis provides compelling data that KA dietary practices and the economic practices of food traditions vary in EBA Armenia.

### 4.1 Primary and secondary products

We observe strong evidence for the exploitation of meat and dairying, evident at various KA sites examined (Fig 2 and Table 1). Dairying is common at settlements located in mountainous regions, as they provide necessary carbohydrates, fats, and nutrients to survive in harsh environments [39, 112], including the ability to store products such as cheese, yoghurt, butter (ghee), etc. [33, 85]. Although the production of fermented dairy products cannot be chemically confirmed, they would have retained nutritional value [113] and fermented milk is still widely consumed in Armenia and South Caucasus today [114].

The identification of dairy products through lipid residue analyses supports the domestic nature of fauna found at the Kura-Araxes settlements examined and across mountainous zones of Armenia and the South Caucasus; however, while dairying is prevalent in Anatolia (7^th^-3^rd^ millennium BCE), Southwest Asia [35, 71, 85, 87, 113], and in the Caucasus [39], we do not see a high predominance of dairying at EBA KA settlements examined here, as biomolecular analysis of dairy residues constituted 31% (*n* = 36) of the potsherds overall, even though we expect to see an intensification due to the faunal evidence and kill-off patterns of domestic species [29, 33] (S1 Table).

Our results emphasise that a mixed economy of plant processing and animal husbandry were the foundation of KA’s economic structure [1, 7, 19, 32, 75]. As such, faunal patterns associated with the Kura-Araxes ‘package’ primarily suggest domestic species [4], where over 50% of the domestic ungulates (in NISP) comprise sheep, goat, and cattle [79]; i.e., at Köhne Shahar in Iran [14, 75], Chobareti [115] and Natsargora in Georgia [116] (Fig 1). Moreover, the dairying date (c. 3500–2500 BCE) ties well with the Eurasian steppe and its earliest evidence for dairying in the Bronze Age [37, 38], as well as the Chalcolithic and Middle Bronze Age in the Caucasus region (earliest detected cattle milk protein dated to 3776–3651 BCE) [39].

The lipid analyses demonstrate that other commodities were important, however, at Gegharot, Talin Tombs, and Sotk-2, dairy products comprise over 50% of the residues in the potsherds analysed. This points to the importance of dairying over other foodstuffs [39] and is to be expected given the high cattle and caprines ratio [77]. While these pots were most likely used for a variety of purposes, their use in processing dairy products was clearly more favoured at settlements in higher mountainous regions of >1000 m asl (Margahovit, Karnut-1, Talin Tombs, Sotk-2 and Gegharot) than lowland zones (Mokhra-Blur and Shengavit) (Fig 1 and S1 Table). Overall, 56% of potsherds contained ruminant adipose fat residues (Table 1), suggesting a meat-based diet was preferred over dairy products [16, 70]. The identification of ruminant carcass products, primarily at Gegharot, Shengavit, Mokhra-Blur, Margahovit, Sotk-2 and Talin Tombs, confirms that various domestic species and wild game formed an important element of the KA subsistence base, and played an important role in the diet of EBA inhabitants. The faunal evidence from the region indicate that the main wild species hunted during the EBA was red deer (*Cervus elaphus*) at Shengavit and gazelle (*Gazella subgutturosa*) at Gegharot [117].

Previously published coeval human and faunal stable isotope analyses across the neighbouring region of Anatolia indicate a degree of homogeneity in dietary habits comprising terrestrial C_3_-based domestic crops and animal proteins, primarily *Ovis* and *Capra* [72]. In terms of the KA, a uniform dietary pattern persists at various settlements, including Chobareti in Georgia [115] and Mentesh Tepe in Azerbaijan [31]. While the EBA human diet focuses on sheep, goat, and cattle husbandry (milk and meat), the KA economic structure is hypothesized as semi-nomadic and/or pastoralism, largely based on indirect proxies including archaeobotanical and zooarchaeological remains [1, 29, 73–75, 79, 118]. Due to the nature of transhumance pastoralism suggested by scholars, the limited pig (*Sus* scrofa) faunal evidence and low incidence of non-ruminant fat residues in potsherds is expected. However, this is remarkably contrasted at Karnut-1, where the Δ^13^C values are characteristic of non-ruminant adipose sources (i.e., pig) and/or mixed non-ruminant/ruminant adipose sources processed in vessels, comprising 55% of potsherds analysed (Fig 2). Additionally, mixing curves display input of significant non-ruminant sources at Margahovit, Mokhra-Blur, and Shengavit (Fig 2). Surprisingly, these results do not correlate with observed faunal assemblages in the region, where pig remains are present in low proportions [29, 77]. Further investigation is required into the osteological data associated with these sites. A mobile pastoral economy requires movement from mountainous to lowland zones [28]; in contrast, a more sedentary lifestyle is required to maintain pigs. Possibly, due to the environmental conditions, pigs were hunted and/or kept in forested areas [20–23] of the north-eastern part of the Armenian Highland, i.e., at Margahovit and nearby settlements.

Significantly, the δ^13^C values observed for the major fatty acids (*n*-C_16:0_ and *n*-C_18:0_), indicate the grazing and fodder of local environments. Clear differences are seen where Mokhra-Blur and Shengavit display local values in C_3_/C_4_ vegetation, in comparison to mountainous settlements comprising strictly C_3_ vegetation, such as at Gegharot, Margahovit and Karnut-1 (Fig 2 and S1 Table). Studies suggest that EBA communities were structured as transhumant or mobile pastoralists [4, 8, 16, 74, 75, 117]; however, data presented here suggest that the settlements examined were most likely agropastoral and/or sedentary-based [119]. Additional research into herd management and mobility at each settlement can shed more light on the KA economic structure. Furthermore, archaeobotanical data from Sos Höyük in Anatolia confirms this finding, where KA inhabitants were most likely settled agriculturalists who practised mixed farming with localised animal husbandry [119]. Agropastoral communities are more robust [120], as the use of carts and ploughs provided transportation opportunities [4, 76], access to rivers, lake basins, and areas suitable for farming and dairy production to survive harsh winters in mountainous areas.

### 4.2 Low abundance of aquatic commodities

Due to the low recovery and taphonomic bias of fish remains across Kura-Araxes archaeological sites, evidence for the consumption of aquatic resources is lacking, and preservation can be problematic [65]. A plethora of fish species would have been available across the region including *Cyprinidae* and *Salmonidae* [79]. The presence of partial suites of aquatic biomarkers (APAAs and IFAs) in potsherds confirms the processing of aquatic resources by KA inhabitants at Gegharot, Shengavit, Mokhra-Blur, and Margahovit, establishing a range of food resources exploited. This is unsurprising due to the vicinity of river systems and freshwater lakes (Fig 1B), including confirmed aquatic proteins consumption through stable isotopic analysis on bone collagen at Chobareti, a neighbouring site in Georgia [115]. However, the low amount of aquatic processing in potsherds mirrors the low abundance of fish bones found in faunal assemblages. Since APAAs form at >200°C temperatures, it is possible to suggest that not all pots were regularly heated to high temperatures [110, 111], or that aquatic resources were most likely cooked without vessels (e.g., over a fire).

### 4.3 Exploitation of plant processing revealed through organic residues

The direct evidence of plant consumption and processing sheds a new light on subsistence strategies of Kura-Araxes mountainous zones and illustrates the first evidence of processing of leafy plants identified in lipid residues from Armenia and the region of Caucasus. The δ^13^C_16:0_ values (-29.0 to -21.3‰) for plant derived fatty acids, together with the presence of long-chain *n*-alkanes, unequivocally confirm the processing of C_3_ grasses, C_4_ salt marshes, and aquatic/water-stressed plants largely available near rivers and Lake Sevan, confirming the archaeobotanical sources commonly cultivated [71, 79, 80, 115]. More specifically, *P*_aq_ values illustrate that aquatic macrophytes are common near Mokhra-Blur and Shengavit settlements in the Ararat Plain, indicating the processing of aquatic plant species from freshwater rivers and lakes [58, 101]. Both cultivated and foraged plants were most likely cooked in vessels, as attested by the presence of plant lipids characteristic of aquatic macrophytes. Five extracts from Shengavit, Gegharot, Margahovit, and Karnut-1 confirmed the processing of plant leaves or fruits, as the distribution of odd-over-even preference for the C_25:0_-C_31:0_ *n*-alkanes is characteristic of an origin from plant lipids and epicuticular waxes (Fig 3A and 3C) [58, 62, 98].

In addition to the main cereal crops such as wheat and barley, carbonised fruit and nut remains have been recovered and likely represent the diversified plant-based dietary intake consumed and processed in these vessels. Plant lipid distributions are common at Shengavit, Margahovit, and Mokhra-Blur, but underrepresented at Karnut-1, Talin Tombs, Sotk-2 and Gegharot. Plant lipids are poorly mobilised resulting in limited transfer to the potsherd matrix [58, 61], and absorbed lipids are replaced over time, representing a mixture of foodstuffs cooked during the vessel’s use-life [121]. As such, low lipid recoveries from pots from Margahovit, Karnut-1, and Gegharot are likely due to the processing of plant-based food, which is typically tenfold lower than that of meat [121] (S2 Table).

### 4.3 Mixed economy and diverse dietary input

From a reconstructive point of view, it is evident that Kura-Araxes populations facilitated a diverse subsistence economy. Distinct culinary practices across the examined KA settlements, highlight variation in foodways and subsistence practices within the region. A high percentage of ruminant carcass fats is evident across various settlements examined, including the mixing of commodities, primarily focused on a high caloric intake of meat consumption, occasional vegetable, and plant-based sources concurrent with secondary products. The use of plant-based sources likely enhanced taste [63]. Evidence of mixing of commodities (Fig 2) suggests boiling, stewing, and steaming foodstuffs, confirming interpretations made through archaeological [16, 19] and ethnoarchaeological contexts of the region [82]; however, it is imperative to note that lipids can be preserved and mixed over time through multiple cooking events [121]. Much like pottery production, food preparation entails similar tasks of ingredient gathering, processing, and mixing [18]; such tasks embody the human behavioural component of cultural transmission [122]. Human-environment interactions with animals and plants encouraged the construction of cultural human behaviour in the past [123], where commensality and food practices are intrinsically tied to social human behaviour, as well as shared identities [17, 18, 124]. Through the biomolecular lipid analysis of KA potsherds, we have shown that culinary practices and food traditions of the KA vary site-by-site in Armenia, one of the regional ‘core’ areas of this cultural phenomenon.

## 5. Conclusions

The results reported here provide the first lipid residue analysis of potsherds from the South Caucasus region, an outlook of dietary practices from multiple sites in Armenia. Our findings report the diversity of culinary and dietary practices across KA settlements and place the earliest evidence of dairying in Armenia and the South Caucasus region (c. 3500–2500 BCE), which is integral to our understanding of cultural evolution of prehistoric people residing in mountainous zones, including the interplay and relationships with neighbouring regions. While the δ^13^C values of the major fatty acids (*n*-C_16:0_ and *n*-C_18:0_) in relation to faunal and archaeobotanical data provide evidence for the exploitation of secondary products, meat and plant processing, the varied sources consumed across settlements suggests that each settlement is structured based on a myriad of local socio-economic practices. The diversity of diet and resource utilisation suggest that these societies were non-hierarchical and community-based [14–16].

Considering the high preservation of lipids at archaeological sites in Armenia, the ongoing investigations into organic materials will likely shed light on many theoretical debates discussed by archaeologists over the past decades. Despite the limitation of chronological data, new methodologies in ^14^C dating can be applied to absorbed lipids in pottery to provide chronological resolution [41, 42]. Lipid residue analysis as a methodological focus has wider implications for prehistoric research, principally in how we define past cultures and lifeways, and is paramount to the holistic approach in synthesising material assemblages to reconstruct past human behavioural systems [17, 125]. The high abundance of animal foodstuffs detected in pottery, alongside plant and aquatic evidence, reflects the importance of both animals and plants in the diet, indicating a mixed economy, agro-pastoral and sedentary-based inhabitation. Compared with faunal, archaeobotanical and environmental data within and from neighbouring regions, our results will facilitate a new understanding into the socio-economic practices of the KA and the reconstruction of dietary practices, including the processing of various foodstuffs. Perhaps variability in foodways and interregional diverse socio-economic practices marked by intensified farming caused its daunting territorial expansion.

## Supporting information

S1 Checklist (DOCX)

S1 Fig Boxplots of the total lipid extract (TLE) concentration from each archaeological site. Results reported in log10 scale (performed in RStudio version 1.2.5033, package ggplot2) [126], exhibiting the concentration of lipids preserved in archaeological vessels from Gegharot, Karnut-1, Margahovit, Mokhra-Blur, Shengavit, Sotk-2 and Talin Tombs.
(TIF)

S2 Fig Mass chromatograms illustrating the presence of APAAs. Mass chromatograms of a) *m/z* 105, b) *m/z* 290, c) *m/z* 318 and d) *m/z* 346 of the acid-extracted FAME from Shengavit (SH68) illustrating the presence of C_18_, C_20_ APAAs and traces of C_22_ APAAs.
(TIF)

S3 Fig Vegetation map of Armenia. Salt marsh vegetation distribution approximated in dashed red lines [88–90]. Sites: (1) Karnut-1, (2) Gegharot, (3) Margahovit, (4) Talin Tombs, (5) Mokhra-Blur, (6) Shengavit, and (7) Sotk-2. Maps modified using Adobe Illustrator by NM. Maps produced by Lily Manoukian using Geosoft Inc. Oasis Montaj (Version 2021.2.1 [20220125–11]) (www.seequent.com); vegetation layer GIS base map source: https://sustainable-caucasus.unepgrid.ch/layers/geonode_data:geonode:vegetation_arm. Reprinted from GEORISK Scientific Research Company under a CC BY license, with permission from Suren Arakelyan, GEORISK Scientific Research Company, original copyright June 1, 2017.
(TIF)

S1 Table Summary of Kura-Araxes archaeological site characteristics. The periodisation of the KA horizon in Armenia includes two temporal phases. KA Phase I (Elar-Aragats) ranges from 3600/3500 to 2900 BCE while KA Phase II (Karnut-Shengavit) ranges from 2900 to 2600/2500 BCE [2, 77].
(DOCX)

S2 Table List of pottery sherds selected for lipid analysis and data (GC, GC-MS and GC-C-IRMS). Sherds were extracted using an established protocol—acidified methanol extraction [84]. Key: (C_n:x_)–carboxylic acids with carbon length n and number of saturations x, SFA–saturated fatty acid, UFA–unsaturated fatty acids, Diacid –*α*, *ω*-dicarboxylic acids, Alk–alkane, Alc–alkanol/alcohol, Ketones, diHFA–dihydroxy fatty acid, APAA– *ω*-(o-alkylphenyl) alkanoic acids, br–branched chain acids dominated by *iso* and *anteiso* C_15_ and C_17_, Isoprenoid fatty acids (IFA): TMTD– 4,8,12-trimethyltridecanoic acid, pris–pristanic acid, phy–phytanic acid; cholesterol, diterpenoids—dehydroabietic acid and other derivatives; terpenoids–indicate the presence of one or several terpenes, including birch bark tar derivatives, and tr–traces. TMS denotes the trimethylsilyl ester and ME denotes methylated. ND is not detected (low concentration).
(DOCX)

S3 Table Plant lipid calculations. P/S ratios, CPI, *P*_aq_, and classification of trimethylsilylated TLEs. P/S ratio = relative abundance ratio of C_16:0_/C_18:0_ fatty acids, where values greater than 4 indicate a plant origin. CPI = measures the relative abundance of odd-over-even carbon chain lengths; CPI values for all plant species have strong odd-chain preferences, ranging between 1.6 and 82.1 [99, 100, 127]. *P*_aq_ = emergent and non-emergent aquatic macrophyte input; *P*_aq_ <0.1 corresponds to a terrestrial plant input; *P*_aq_ 0.1–0.4 to emergent macrophytes, and *P*_aq_ 0.4–1.0 to submerged or floating macrophytes [101]. N/D–not determined, signal intensity too low.
(DOCX)
