# Supplementary figures and images for: Diverse dietary practices across the Early Bronze Age ‘Kura-Araxes culture’ in the South Caucasus
Source: PLoS One. 2022 Dec 21;17(12):e0278345. doi: 10.1371/journal.pone.0278345 (PMC9770345; doi:10.1371/journal.pone.0278345)

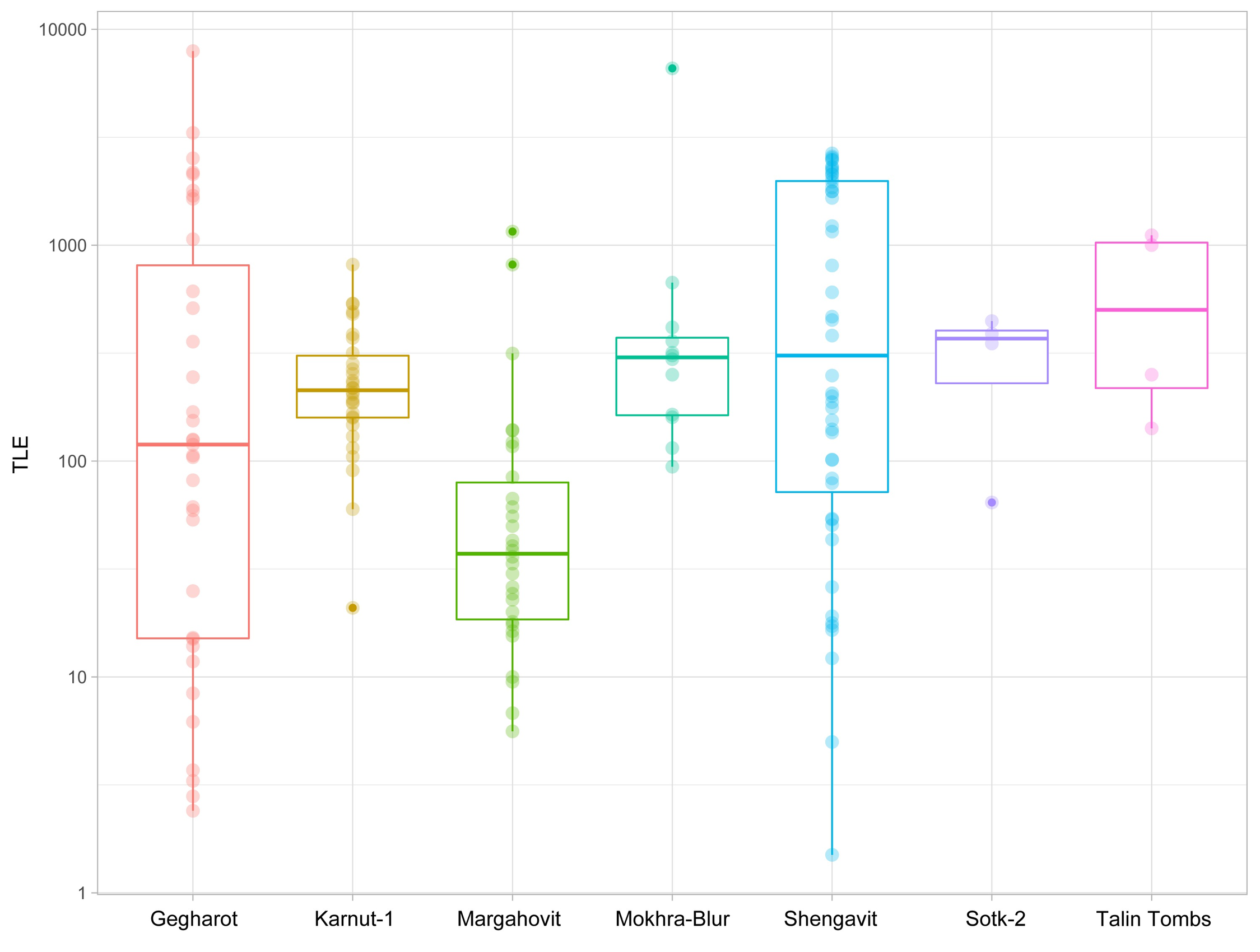

Supplement: S1 Fig — Results reported in log10 scale (performed in RStudio version 1.2.5033, package ggplot2) [126], exhibiting the concentration of lipids preserved in archaeological vessels from Gegharot, Karnut-1, Margahovit, Mokhra-Blur, Shengavit, Sotk-2 and Talin Tombs. (TIF) [file pone.0278345.s002.tif]

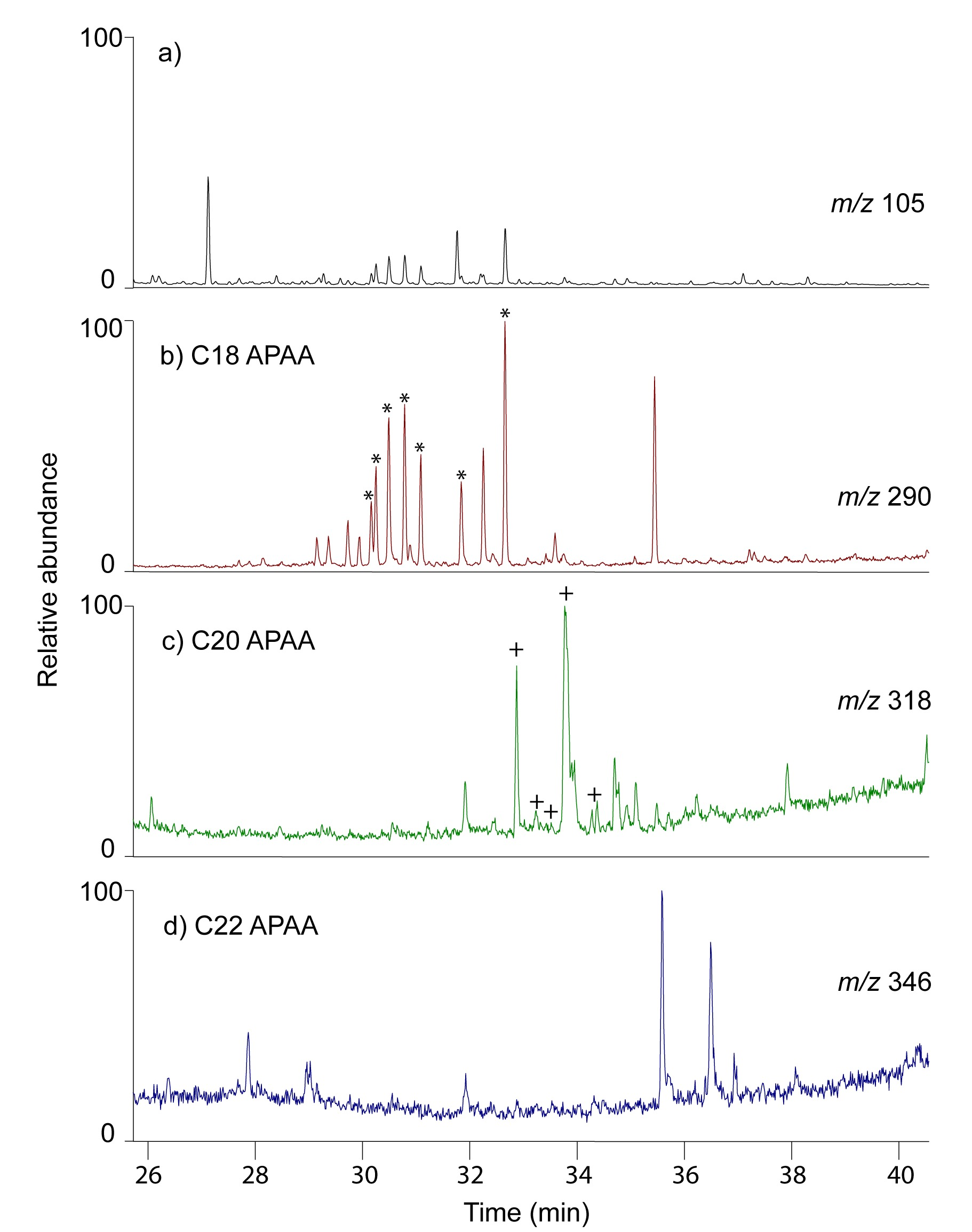

Supplement: S2 Fig — Mass chromatograms of a) m/z 105, b) m/z 290, c) m/z 318 and d) m/z 346 of the acid-extracted FAME from Shengavit (SH68) illustrating the presence of C18, C20 APAAs and traces of C22 APAAs. (TIF) [file pone.0278345.s003.tif]

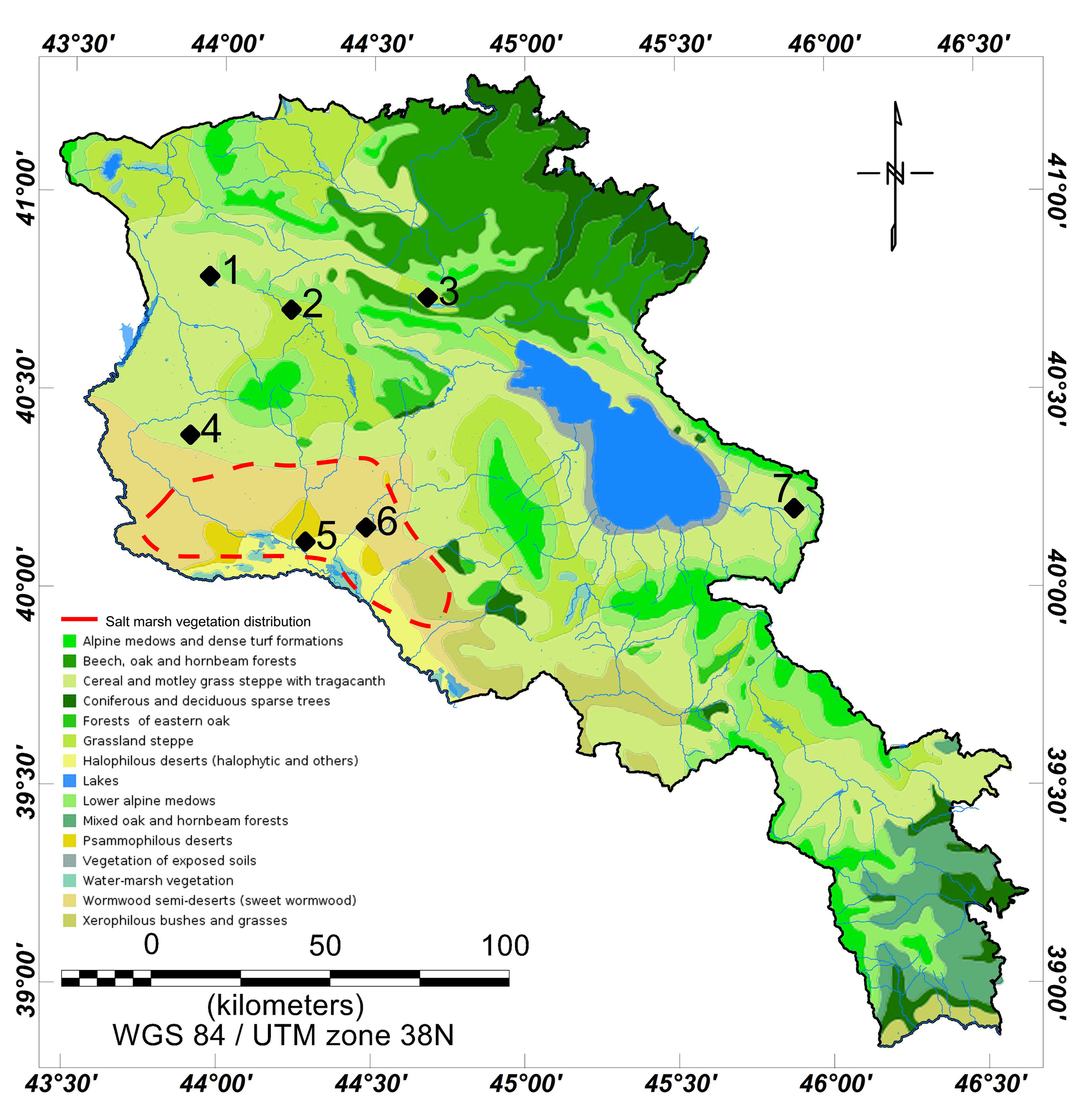

Supplement: S3 Fig — Salt marsh vegetation distribution approximated in dashed red lines [88–90]. Sites: (1) Karnut-1, (2) Gegharot, (3) Margahovit, (4) Talin Tombs, (5) Mokhra-Blur, (6) Shengavit, and (7) Sotk-2. Maps modified using Adobe Illustrator by NM. Maps produced by Lily Manoukian using Geosoft Inc. Oasis Montaj (Version 2021.2.1 [20220125–11]) (www.seequent.com); vegetation layer GIS base map source: https://sustainable-caucasus.unepgrid.ch/layers/geonode_data:geonode:vegetation_arm. Reprinted from GEORISK Scientific Research Company under a CC BY license, with permission from Suren Arakelyan, GEORISK Scientific Research Company, original copyright June 1, 2017. (TIF) [file pone.0278345.s004.tif]
