# Supplementary material for: Diverse dietary practices across the Early Bronze Age ‘Kura-Araxes culture’ in the South Caucasus
Source: PLoS One. 2022 Dec 21;17(12):e0278345. doi: 10.1371/journal.pone.0278345 (PMC9770345; doi:10.1371/journal.pone.0278345)
Supplement: S1 Table — The periodisation of the KA horizon in Armenia includes two temporal phases. KA Phase I (Elar-Aragats) ranges from 3600/3500 to 2900 BCE while KA Phase II (Karnut-Shengavit) ranges from 2900 to 2600/2500 BCE [2, 77]. (DOCX) [file pone.0278345.s005.docx]

# **S1 Table. Summary of Kura-Araxes archaeological site characteristics.**

The periodization of the KA horizon in Armenia includes two temporal phases. KA Phase I (*Elar-Aragats*) ranges from 3600/3500 to 2900 BCE while KA phase II (*Karnut-Shengavit*) ranges from 2900 to 2600/2500 BCE (Badalyan, 2014; Badalyan et al., 2008).

| **Site** | **Potsherds analysed** | **Longitude** | **Latitude** | **Altitude (m asl)** | **Radiocarbon date (cal. B.C.E)** | **Pottery** | **Settlement type** | **Archaeobotany** | **Faunal management strategy** | **References and notes** |
| --- | --- | --- | --- | --- | --- | --- | --- | --- | --- | --- |
| Gegharot | 35 | 44.225278 | 40.705833 | 2155 | KA I-II | Black-burnished | Mountainous - Settlement and fortress | Cereals, barleys (hulled barleys, hulled 2-row barley, 6-row barleys, hulled 6-row barley); wheat (tetra- and/or hexaploid wheats [naked and/or hulled], naked wheats, naked bread wheat, hulled wheats, spelt type wheat, emmer, einkorn type); other cereals (rye), and pulses (lentil, pea, bitter vetch, grass pea, vetches, [wild?]); oil-crops, flax(?); evidence of grape, *Rosa* sp and *Rubus* sp. | Meat-based: diversity of sheep/goat (47.4%), and cattle (49%); occasional wild animals: deer, gazelle, wolf(possibly), toad, snake, and birds.  KA I = absence of pigs; KA II = presence of pigs (low proportions) | Monahan, 2007; Badalyan et al., 2008; Badalyan et al., 2010; Badalyan et al., 2014; Hovsepyan, 2015; Chahoud et al., 2015; Jude et al., 2010. |
| Shengavit | 48 | 44.476667 | 40.157222 | 923 | KA I-II | Black, buff, red-burnished | Flat, lowland – settlement and necropolis | Cereal-based; large quantities of *Triticum sphaerococcum*; barleys (hulled barleys, hulled 2-row barleys, 2-row barleys, 6-row barleys, hulled 6-row barleys, naked barleys(?); wheat (tetra- and/or hexaploidy wheats [naked and/or hulled], naked wheats, naked bread wheat, macaroni wheat(?), emmer); pulses/vetches (wild?); oil-crops: flax(?); grape; *Rosa* sp and *Rubus* sp. | Meat-based; sheep/goat and cattle husbandry; horse, dog and pig (rare); wild game: red and roe deer | Badalyan et al., 2014; Piro and Crabtree, 2017; Hovsepyan, 2015; Chahoud et al., 2015; see Simonyan and Rothman, 2015, for radiocarbon dates. |
| Mokhra-Blur | 13 | 44.277694 | 40.120111 | 844 | KA II | Black-burnished | Flat/hill, lowland - settlement | Cereal-based; wheat and barley; Nutlets of *Lithospermum arvense*. | Meat-based; sheep/goat and cattle husbandry; horse, dog and pig (rare). | Badalyan et al., 2014; Piro and Crabtree, 2017; Badalyan.et al., 2008; Hovsepyan, 2015; Badalyan and Avetisyan, 2007; Chahoud et al., 2015. |
| Sotk-2 | 4 | 45.886111 | 40.203333 | 2100 | KA I-II | Black-burnished | Mountainous – lake – settlement; steppic environment | Cereal-based; wheat (*Triticum*) and barley (*Hordeum*); (hulled barleys), wheat (tetra- and/or hexaploidy wheats [naked and/or hulled], naked wheats), vetches (wild?).; evidence of *Rosa* sp and *Rubus* sp. | Meat-based: cattle, sheep/goat; few horses, deer, pig, and wolf/dog | Hovsepyan, 2015. |
| Talin Tombs | 4 | 43.895000 | 40.387361 | 1600 | KA I | Black, buff-burnished | Mountainous – settlement and necropolis (“cult” enclosure) | Cereal-based? | Meat-based: cattle, sheep, goat, horse (one tooth), and wild species | Badalyan and Avetisyan, 2007. |
| Karnut-1 | 30 | 43.953942 | 40.788686 | 1600 | KA II | Black-burnished | Mountainous - settlement | Cereal-based? | Meat-based; Cattle, sheep/goat, horses, and deer. | Badalyan and Avetisyan, 2007; archaeozoological reports, unpublished. |
| Margahovit | 30 | 44.676667 | 40.738667 | 1850 | KA II | Black. buff-burnished | Fortified settlement atop hill | Cereal—31%, (Triticeae gen. spp.); Wheat—86%, (*Triticum* spp.); barley and wheat—14% (*Hordeum vulgare*); (hulled barleys, hulled 6-row barley); wheat (tetra- and/or hexaploidy wheats [naked and/or hulled], naked wheats, naked bread wheat, emmer); ryes, and vetches; Weeds—28% (Poaeceae, Fabaceae, Rubiaceae, Boraginaceae, Polygonaceae, Cyperaceae, Brassicaceae, Chenopodiaceae, Ranunculaeceae, Violaceae, Lamiaceae). | Meat-based: cattle (38%-74%), sheep/goat (10-17%), pig (4-12%), deer (7-17%), mouflon (2-10%), European roe deer (1-2%), and brown bear (2-5%). | Gevorgyan et al., 2021; Hovsepyan, 2015. |

**References for Supplementary Table (S1)**

Badalyan R. New data on the periodization and chronology of the Kura-Araxes culture in Armenia. Paléorient. 2014;40(2), 71-92.

Badalyan R, Avetisyan P. Bronze and Early Iron Age Archaeological Sites in Armenia I. Mt. Aragats and its surrounding region. British Archaeological Report 1697, Oxford; 2007.

Badalyan R, Smith AT, Lindsay I, Khatchadourian L, Avetisyan P. Village, fortress, and town in Bronze and Iron Age South Caucasia: a preliminary report on the 2003-2006 investigations of project ArAGATS on the Tsaghkahovit Plain, Republic of Armenia. Archäologische Mitteilungen aus Iran und Turan 40; 2008. pp. 45-105.

Badalyan R, Smith AT, Khatchadourian L. Project ArAGATS: 10 years of investigations into Bronze and Iron Age sites in the Tsaghkahovit Plain, Republic of Armenia. Türkiye Bilimler Akademisi Arkeoloji Dergisi 13; 2010. pp. 263-276.

Badalyan R, Smith AT, Lindsay I, Harutyunyan A, Greene A, Marshall M, et al. A preliminary report on the 2008, 2010, and 2011 investigations of Project ArAGATS on the Tsaghkahovit Plain, Republic of Armenia. Archäologische Mitteilungen aus Iran und Turan, Band 46; 2014. pp. 149-222.

Gevorgyan A, Danielyan H, Vanyan H, Hovsepyan R, Manaseryan N, Manoukian N, et al. Margahovit: craft and subsistence economy in a Bronze/Iron Age community between Pambak and Bazum Mountains, Armenia. In: Avetisyan P, Bobokhyan A, editors. Archaeology of Armenia in Regional Context. Publishing House of the Institute of Archaeology and Ethnography, Yerevan; 2021. pp. 69-88.

Hovsepyan R. On the agriculture and vegetal food economy of Kura-Araxes Culture in the South Caucasus. Paléorient. 2015;41, 69-82.

Jude F, Marguerie D, Badalyan R, Smith AT, Delwaide A. Wood resource management based on charcoals from the Bronze Age site of Gegharot (central Armenia). Quaternary International. 2016;395, 31-44.

Monahan BH. Nomadism in the Early Bronze Age Southern Caucasus: The Faunal Perspective. In: Popova L, Hartley C, Smith AT, editors. Social Orders and Social Landscapes: Proceedings of the 2005 University of Chicago Conference on Eurasian Archaeology. Cambridge Scholars Press, Cambridge; 2007. pp. 379-292.

Piro JJ, Crabtree PJ. Zooarchaeological evidence for pastoralism in the Early Transcaucasian Culture. In: Mashkour M. Beech M, editors. Archaeozoology of the Near East 9: in honour of Hans-Peter Uerpmann and Francois Poplin. Oxbow Books, Oxford; 2017. pp. 273-283.

Simonyan H, Rothman MS. Regarding ritual behaviour at Shengavit, Armenia. ANES. 2015;52, 1-46.
