# Supplementary material for: Diverse dietary practices across the Early Bronze Age ‘Kura-Araxes culture’ in the South Caucasus
Source: PLoS One. 2022 Dec 21;17(12):e0278345. doi: 10.1371/journal.pone.0278345 (PMC9770345; doi:10.1371/journal.pone.0278345)
Supplement: S2 Table — Sherds were extracted using an established protocol—acidified methanol extraction [84]. Key: (Cn:x)–carboxylic acids with carbon length n and number of saturations x, SFA–saturated fatty acid, UFA–unsaturated fatty acids, Diacid –α, ω-dicarboxylic acids, Alk–alkane, Alc–alkanol/alcohol, Ketones, diHFA–dihydroxy fatty acid, APAA– ω-(o-alkylphenyl) alkanoic acids, br–branched chain acids dominated by iso and anteiso C15 and C17, Isoprenoid fatty acids (IFA): TMTD– 4,8,12-trimethyltridecanoic acid, pris–pristanic acid, phy–phytanic acid; cholesterol, diterpenoids—dehydroabietic acid and other derivatives; terpenoids–indicate the presence of one or several terpenes, including birch bark tar derivatives, and tr–traces. TMS denotes the trimethylsilyl ester and ME denotes methylated. ND is not detected (low concentration). (DOCX) [file pone.0278345.s006.docx]

# **S2 Table. List of pottery sherds selected for lipid analysis and data (GC, GC-MS and GC-C-IRMS).**

List of pottery sherds selected for lipid analysis (GC-MS, GC-C-IRMS). Sherds were extracted using an established protocol—acidified methanol extraction (Correa-Ascencio and Evershed, 2014).

Key: (C_n:x_) – carboxylic acids with carbon length n and number of saturations x, SFA – saturated fatty acid, UFA – unsaturated fatty acids, Diacid –$\alpha$,$\omega$-dicarboxylic acids, Alk – alkane, Alc – alkanol/alcohol, Ketones, diHFA – dihydroxy fatty acid, APAA – $\omega$-(o-alkylphenyl) alkanoic acids, br – branched chain acids dominated by *iso* and *anteiso* C_15_ and C_17_, Isoprenoid fatty acids (IFA): TMTD – 4,8,12-trimethyltridecanoic acid, pris – pristanic acid, phy – phytanic acid; cholesterol, diterpenoids - dehydroabietic acid and other derivatives; terpenoids – indicate the presence of one or several terpenes, including birch bark tar derivatives, and tr – traces. TMS denotes the trimethylsilyl ester and ME denotes methylated. ND is not detected (low concentration).

| **Sample** | **Site** | **Contextual information** | **Lipid concentration (ug g^-1^)** | **Total lipid in extract (ug)** | **δ^13^C_16:0_**  **(‰)** | **δ^13^C_18:0_**  **(‰)** | **Δ^13^C**  **(‰)** | **Classification** | **Compounds (including aquatic biomarkers)** |
| --- | --- | --- | --- | --- | --- | --- | --- | --- | --- |
| **G3** | Gegharot | T30-101-EB2 | 2.83 | 8.37 | - | - | - | - | - |
| **G4** | Gegharot | T30-101-EB2 | 1.10 | 3.30 | - | - | - | - | - |
| **G5** | Gegharot | T30-101-EB2 | 3384.9 | 7926.0 | -27.1 | -30.4 | -3.4 | Dairy | SFA (C_14:0-28:0_), UFA (C_16:1, 18:1, 22:1_), Alc (C_18, 26_) |
| **G6** | Gegharot | T30-101-EB2 | 782.7 | 2130.6 | -26.6 | -31.6 | -5.1 | Dairy | SFA (C_15:0-28:0, 30:0_), UFA (C_16:1, 18:1, 22:1_), Alc (C_16, 18, 20-22, 24, 25, 28, 30_), Alk (C_22, 27, 28, 33_), $\omega$-Hydroxy FAME (C_22_), Hydroxy FAME (C_24_) |
| **G7** | Gegharot | T30-101-EB2 | 0.99 | 2.78 | - | - | - | - | - |
| **G8** | Gegharot | T30-101-EB2 | 180.3 | 510.9 | -26.5 | -31.1 | -4.5 | Dairy | SFA (C_14:0-28:0_), UFA (C_16:1, 18:1, 22:1_), Alc (C_16, 18, 20- 22, 24, 26, 27_), Alk (C_19, 28_), Hydroxy FAME (C_18_) |
| **G9** | Gegharot | T30-101-EB2 | 971.9 | 2525.6 | - | - | -- | - | - |
| **G10** | Gegharot | T30-101-EB2 | 359.9 | 1065.6 | -26.7 | -31.6 | -4.9 | Dairy | SFA (C_14:0-28:0, 30:0_), UFA (C_16:1, 18:1_), Br (C_17_), Alc (C_14, 16-20, 22-24, 26, 28, 30_) |
| **G11** | Gegharot | T30-101-EB2 | 295.3 | 611.4 | -27.5 | -32.0 | -4.5 | Dairy | SFA (C_14:0-26:0, 28:0, 30:0_), UFA (C_16:1, 18:1, 22:1_), Br (C_17_), Alc (C_14, 18, 24, 26, 28, 30_), Alk (C_29, 31_) Diacid (C_14, 16_) |
| **G12** | Gegharot | T36-3-EB2 | 5.00 | 13.89 | - | - | - | - | - |
| **G13** | Gegharot | T36-3-EB2 | 9.81 | 25.0 | -27.2 | -29.7 | -2.5 | Ruminant adipose fats | SFA (C_16:0-26:0, 28:0, 30:0_), UFA (C_16:1, 18:1, 20:1, 22:1_), Alc (C_14, 16-18, 20-22, 24, 26_), Alk (C_29, 31_) |
| **G14** | Gegharot | T36-3-EB2 | 5.25 | 15.0 | -26.8 | -29.7 | -3.0 | Ruminant adipose fats? | SFA (C_16:0-18:0, 20:0, 26:0, 28:0, 30:0_), UFA (C_18:1, 22:1_), Alc (C_16, 18_), Alk (C_29, 31, 33_) |
| **G15** | Gegharot | T36-3-EB2 | 96.1 | 244.9 | -25.8 | -27.8 | -2.0 | Ruminant adipose fats | SFA (C_14:0-26:0_), UFA (C_16:1, 18:1, 22:1_), Alc (C_18, 24, 26_) |
| **G16** | Gegharot | T36-3-EB2 | 646.4 | 1694.6 | -26.9 | -28.4 | -1.5 | Ruminant adipose fats | SFA (C_16:0-26:0_), UFA (C_18:1, 22:1_), Alc (C_16-22, 24, 26_), Hydroxy FAME (C_24-26_), Chol |
| **G170** | Gegharot | T36-3-EB2 | 4.00 | 11.8 | - | - | - | - | SFA (C_16:0-18:0, 20:0-24:0,_ _26:0_), UFA (C_16:1, 18:1, 22:1_), Alc (C_14-18, 20-22, 24, 26_) |
| **G171** | Gegharot | T36-3-EB2 | 0.87 | 2.40 | - | - | - | - | - |
| **G18** | Gegharot | T36-3-EB2 | 2.23 | 6.20 | - | - | - | - | SFA (C_16:0, 18:0, 23:0-26:0_), UFA (C_18:1, 22:1_), Alc (C_16, 18_), Hydroxy FAME (C_24_) |
| **G19** | Gegharot | T36-3-EB2 | 603.4 | 1639.8 | -26.0 | -30.5 | -4.4 | Dairy | SFA (C_14:0-26:0, 28:0, 30:0_), UFA (C_18:1, 22:1_), Br (C_15, 17_), Hydroxy FAME (C_24_) |
| **G20** | Gegharot | T36-3-EB2 | 812.6 | 2173.6 | - | - | - | - | - |
| **G21** | Gegharot | T36-3-EB2 | 59.1 | 125.9 | -26.9 | -29.7 | -2.8 | Ruminant adipose fats | SFA (C_15:0-26:0, 28:0_), UFA (C_16:1, 18:1, 20:1, 22:1_), Br (C_17_) Alc (C_16, 18, 20, 22, 24, 26_), Alk (C_30_), $\omega$-Hydroxy FAME (C_24_), Hydroxy FAME (C_24_), Chol |
| **G22** | Gegharot | T36-3-EB2 | 21.2 | 59.1 | -26.8 | -31.0 | -4.2 | Dairy | SFA (C_15:0-30:0_), UFA (C_18:1, 22:1_), Br (C_17_) Alk (C_31, 33_), Hydroxy FAME (C_24_) |
| **G23** | Gegharot | T36-3-EB2 | 6.20 | 15.2 | -27.0 | -28.4 | -1.4 | Ruminant adipose fats | SFA (C_16:0-24:0, 28:0, 30:0_), UFA (C_18:1, 22:1_), Alc (C_18-20, 22, 24_), $\omega$-Hydroxy FAME (C_24_), Hydroxy FAME (C_22-25_), Acetic acid |
| **G24** | Gegharot | T36-3-EB2 | 652.3 | 1790.3 | -26.7 | -31.9 | -5.2 | Dairy | SFA (C_15:0-28:0, 30:0_), UFA (C_18:1, 22:1_), Alc (C_16, 18, 22, 24, 26, 28, 30_), Hydroxy FAME (C_23?, 24_), Ketones (C_29?, 31, 33_) |
| **G25** | Gegharot | T36-3-EB2 | 1.52 | 3.73 | - | - | - | - | - |
| **G26** | Gegharot | T36-3-EB2 | 42.1 | 119.2 | -25.9 | -31.4 | -5.5 | Dairy | SFA (C_15:0-26:0_, _28:0, 30:0_), UFA (C_16:1, 18:1, 22:1_), Alc (C_16, 18, 20, 22, 24, 26, 30_), $\omega$-Hydroxy FAME (C_24_), Hydroxy FAME (C_23-26_) |
| **G27** | Gegharot | Ge.18.T39.31 | 19.3 | 53.5 | - | - | - | - | SFA (C_15:0, C16:0, 18:0, 24:0, 26:0, 28:0_), UFA (C_16:1, C18:1_), Br (C_17_) |
| **G28** | Gegharot | Ge.18.T39.31 | 35.1 | 104.2 | -27.4 | -30.3 | -2.9 | Ruminant adipose fats and plants wax | SFA (C_14:0-20:0, 22:0-26:0, 28:0, 30:0_), UFA (C_16:1, 18:1_), Alc (C_16, 18, 20, 22, 26, 28_), Diacid (C_18, 20, 22, 24_), $\omega$-Hydroxy FAME (C_20, 22, 24_), Hydroxy FAME (C_24, 26_), terpenoids: Lup-2,20(29)-dien-28-ol (TMS), Allobetul-2-ene, 28-oxoallobetul-2-ene, Betulone (TMS), and Betulin (TMS) |
| **G29** | Gegharot | Ge.18.T39.31 | 34.1 | 81.5 | -24.2 | -25.3 | -1.1 | Ruminant adipose fats | SFA (C_15:0-20:0, 22:0-26:0, 28:0-30:0, 32:0_), UFA (C_16:1, C18:1_), Br (C_15, 17_), Alc (C_16-18, 24, 26, 30_), $\omega$-Hydroxy FAME (C_22, 24_), Hydroxy FAME (C_16, 19, 24_), Ketone (C_31, 33?_) |
| **G30** | Gegharot | Ge.18.T39.31 | 53.4 | 153.9 | - | - | - | - | - |
| **G31** | Gegharot | Ge.18.T39.31 | 1285.8 | 3313.3 | -26.6 | -30.4 | -3.8 | Dairy, plant wax and aquatic(?) | SFA (C_14:0-24:0, 26:0_), UFA (C_18:1_), Br (C_17_), Diacids (C_20, 22_),$\omega$-Hydroxy FAME (C_22_), terpenoids: Lup-2,20(29)-dien-28-ol (TMS), IFAs (TMTD, Phy, Pris) |
| **G32** | Gegharot | Ge.18.T39.31 | 142.5 | 357.4 | -27.3 | -30.9 | -3.6 | Dairy | SFA (C_14:0-26:0, 28:0-30:0, 32:0_), UFA (C_18:1_), Alc (C_18, 24, 26, 28, 30_), Diacid (C_22, 24, 26, 28_), $\omega$-Hydroxy FAME (C_22, 24_), Sulfur, IFA: phy |
| **G33** | Gegharot | Ge.18.T39.31 | 50.1 | 125.0 | -27.3 | -29.4 | -2.1 | Ruminant adipose fats | SFA (C_16:0, 18:0-20:0, 24:0, 26:0_) |
| **G34** | Gegharot | Ge.18.T39.31 | 36.1 | 106.1 | - | - | - | - | SFA (C_16:0, 18:0, 24:0_), UFA (C_16:1, 18:1_) |
| **G35** | Gegharot | Ge.18.T39.31 | 56.9 | 168.7 | -26.8 | -32.1 | -5.4 | Dairy | SFA (C_16:0-18:0, 20:0-26:0, 28:0, 30:0_), UFA (C_18:1_), Alc (C_16, 18, 20, 24, 26, 28, 30_), Alk (C_21, 23, 25-27, 29, 31, 33_), Hydroxy FAME (C_24-26_), IFA: phy |
| **G36** | Gegharot | Ge.18.T39.31 | 21.7 | 61.3 | -26.3 | -29.1 | -2.8 | Ruminant adipose fats | SFA (C_16:0-18:0, 24:0-26:0_), UFA (C_18:1_), Alc (C_16, 18, 26_) |
| **SHA** | Shengavit | Trench K6, Locus 1027 | 174.8 | 466.8 | -28.5 | -27.9 | 0.6 | Non-ruminant adipose fats | SFA (C_14:0, 16:0, 18:0, 20:0_), Alk (C_20, 22, 23_) |
| **SHEN10** | Shengavit | Trench K6, Locus 1107 | 268.2 | 604.8 | -26.1 | -28.4 | -2.3 | Ruminant adipose fats | SFA (C_15:0-22:0_), UFA (C_18:1_), Alc (C_14, 18_) |
| **SHEN26** | Shengavit | Trench K6, Locus 1087 | 847.6 | 2492.9 | -27.6 | -28.9 | -1.3 | Ruminant adipose fats | SFA (C_14:0, 16:0-20:0, 22:0, 24:0_), UFA (C_18:1_), Alc (C_12, 14, 16, 18, 20_), Alk (C_17-22_), Hydroxy FAME (C_12_) |
| **SH10** | Shengavit | Trench M5, Locus 24028 | 1101.5 | 2661.5 | -25.8 | -28.6 | -2.8 | Ruminant adipose fats | SFA (C_14:0-30:0_), UFA (C_16:1, 18:1_), Alc (C_14, 16, 18, 20-24, 26, 27, 30_), Alk (C_23_), Hydroxy FAME (C_10_), Chol |
| **SH11** | Shengavit | Trench K6, Locus 1012 | 80.0 | 135.7 | -24.5 | -25.4 | -0.8 | Ruminant adipose fats | SFA (C_14:0-20:0, 22:0, 23:0, 26:0_), UFA (C_16:1, 18:1_), Alc (C_16, 18, 20_), Alk (C_18_) |
| **SH12** | Shengavit | Trench M5, Locus 24028 | 1.73 | 4.96 | - | - | - | - | - |
| **SH13** | Shengavit | Trench K6, Locus 1123 | ND | ND | - | - | - | - | - |
| **SH14** | Shengavit | Trench K6, Locus 1123 | 0.1 | 0.2 | - | - | - | - | - |
| **SH15** | Shengavit | Trench K6, Locus 1125 | 1.6 | 2.2 | - | - | - | - | - |
| **SH23** | Shengavit | Trench K6, Locus 1108 | 774.3 | 2204.4 | -26.7 | -29.1 | -2.3 | Ruminant adipose fats | SFA (C_15:0-26:0_), UFA (C_16:1, 18:1_), Alc (C_16-20, 26_), Alk (C_20, 23, 25, 26, 29_) |
| **SH27** | Shengavit | Trench K6, Locus 1029 | 733.5 | 1773.9 | -27.3 | -29.0 | -1.7 | Ruminant adipose fats | SFA (C_14:0-18:0, 24:0_), UFA (C_16:1, 18:1_), Alc (C_14-22, 24_), Alk (C_20, 22-24, 26_), Cholesterol |
| **SH30** | Shengavit | Trench K6, Locus 1101 | 936.9 | 2576.9 | - | - | - | - | - |
| **SH32** | Shengavit | Trench K6, Locus 1020 | 17.0 | 43.3 | -25.9 | -27.5 | -1.6 | Ruminant adipose fats and plant | SFA (C_16:0-18:0, 20:0-28:0, 30:0_), UFA (C_16:1, 18:1_), Alc (C_14, 16-18, 20, 22, 24, 26, 28, 30_), Alk (C_19-23, 25-31, 33_), unidentified terpenoids |
| **SH35** | Shengavit | Trench K6, Locus 1020 | 710.2 | 1655.6 | - | - | - | - | - |
| **SH37** | Shengavit | Trench K6, Locus 1056 | 0.64 | 1.48 | - | - | - | - | - |
| **SH38** | Shengavit | Trench K6, Locus 1056 | 109.8 | 248.9 | -25.8 | -27.4 | -1.6 | Ruminant adipose fats | SFA (C_16:0-21:0_), UFA (C_18:1_), Br (C_17_) |
| **SH43** | Shengavit | Trench K6, Locus 1125 | 159.8 | 450.3 | -26.1 | -27.4 | -1.3 | Ruminant adipose fats | SFA (C_14:0-21:0_), UFA (C_18:1_) |
| **SH58** | Shengavit | Trench K6, Locus 1014 | 10.4 | 26.1 | - | - | - | - | SFA (C_14:0-18:0_), UFA (C_16:1, 18:1_), Alc (C_16_), Alk (C_19-21_), plasticizers |
| **SH64** | Shengavit | Trench K6, Locus 1069 | 20.7 | 54.1 | -23.9 | -25.8 | -1.9 | Ruminant adipose fats | SFA (C_16:0-26:0, 28:0, 30:0_), UFA (C_18:1_), Alc (C_16, 18, 20-22, 24, 26, 28, 30_), Alk (C_20, 21, 23-25, 27, 29, 31_), Hydroxy FAME (C_24_) |
| **SH67** | Shengavit | Trench K6, Locus 1167 | 737.0 | 2125.4 | - | - | - | - | - |
| **SH79** | Shengavit | Trench M5, Locus 24028 | 849.3 | 2246.2 | -27.0 | -28.9 | -1.8 | Ruminant adipose fats and plant | SFA (C_14:0-18:0, 24:0, 26:0_), UFA (C_16:1,_ _18:1_), Alc (C_14-22, 24, 26_), Alk (C_19-27, 29_), Hydroxy FAME (C_12, 15_) |
| **SH105** | Shengavit | Trench M5, Locus 24004 | 904.0 | 2315.8 | -27.4 | -28.9 | -1.5 | Ruminant adipose fats and plant(?) | SFA (C_15:0-21:0, 23:0-26:0_), UFA (C_18:1_), Alc (C_14-18, 20-22, 24, 26_), Alk (C_18-33_), Cholesterol, unidentified terpenoid |
| **SH110** | Shengavit | Trench M5, Locus 24001 | 821.6 | 2134.2 | -22.5 | -23.0 | -0.6 | Ruminant adipose fats | SFA (C_16:0-26:0_), UFA (C_18:1_) |
| **SH114** | Shengavit | Trench J5, Locus 2081 | 128.2 | 381.4 | -21.3 | -26.4 | -5.1 | Dairy | SFA (C_15:0-26:0_), UFA (C_18:1_), Br (C_17_) |
| **SH115** | Shengavit | Trench J5, Locus 2081 | 794.5 | 1850.9 | - | - | - | - | - |
| **SH116** | Shengavit | Trench J5, Locus 2081 | 7.49 | 19.1 | -27.0 | -27.9 | -0.9 | Ruminant adipose fats | SFA (C_16:0-20:0, 22:0, 24:0, 26:0, 28:0_), UFA (C_18:1_), Alc (C_17, 18, 20, 22, 24, 26_) |
| **SH117** | Shengavit | Trench J5, Locus 2081 | 804.7 | 1966.3 | - | - | - | - | - |
| **SH118** | Shengavit | Trench J5, Locus 2081 | 76.2 | 199.9 | - | - | - | - | - |
| **SH132** | Shengavit | Trench J5, Locus 2072 | 1078.8 | 2536.7 | -26.6 | -28.7 | -2.2 | Ruminant adipose fats and plant wax | SFA (C_16:0-22:0, 24:0, 26:0_), UFA (C_16:1, 18:1_), Alc (C_14, 16-18, 20, 26_), Diacid (C_20, 22_), $\omega$-Hydroxy FAME (C_18, 20, 22_)-Aliphatic diol? (Component of waxes and beeswax), Terpenoids: Lup-2,20(29)-diene, Lup-2,20(29)-dien-28-ol (TMS), Allobetul-2-ene, Betulin (TMS), Betulone (TMS), Betulin (TMS), Allobetulinol (TMS) |
| **SH134** | Shengavit | Trench J5, Locus 2072 | 5.80 | 17.2 | - | - | - | - | - |
| **SHK6** | Shengavit | Trench K6, Locus 1056 | 7.26 | 16.5 | - | - | - | - | SFA (C_16:0, 18:0_), APAA C18? |
| **SHA2** | Shengavit | Trench K6, Locus 1027 | 56.3 | 154.6 | -28.1 | -29.4 | -1.3 | Ruminant adipose fats | SFA (C_14:0, 16:0, 18:0_), Alc (C_12, 14, 16-18_), Alk (C_17-23_), Benzoic acid |
| **SH9** | Shengavit | Trench K6, Locus 1167 | 686.0 | 1226.3 | -25.6 | -23.5 | 2.1 | Non-ruminant adipose fats & aquatic(?) | SFA (C_14:0-30:0_), UFA (C_16:1, 18:1_), Br (C_15, 17_), Alc (C_19_), IFAs: TMTD, pris, phy |
| **SH20** | Shengavit | Trench K6, Locus 1101 | 7.13 | 17.8 | -28.6 | -29.4 | -0.8 | Ruminant adipose fats | FAs C16, 18 |
| **SH21** | Shengavit | Trench K6, Locus 1101 | 19.1 | 50.5 | -26.5 | -29.0 | -2.4 | Ruminant adipose fats and plant | SFA (C_14:0-21:0, 24:0_), Alc (C_16, 18, 26_), Alk (C_18-31_), IFAs: TMTD |
| **SH29** | Shengavit | Trench K6, Locus 1101 | 4.31 | 12.2 | - | - | - | - | SFA (C_16:0, 18:0_) |
| **SH45** | Shengavit | Trench K6, Locus 1027 | 19.3 | 53.6 | -29.0 | -29.3 | -0.3 | Ruminant adipose fats and plant(?) | SFA (C_14:0, 16:0-18:0_), Alc (C_18_), Alk (C_19, 21, 22_), Sulfur |
| **SH47** | Shengavit | Trench K6, Locus 1123 | 51.6 | 140.2 | -25.5 | -27.5 | -2.1 | Ruminant adipose fats | SFA (C_14:0, 16:0-26:0, 30:0_), UFA (C_18:1_), Alc (C_18, 26, 30_), Sulfur |
| **SH48** | Shengavit | Trench M5, Locus 24015 | 951.0 | 2480.0 | -26.4 | -29.9 | -3.5 | Dairy and aquatic(?) | SFA (C_14:0-30:0_), Br (C_17_), APAA C18?, 20(tr), 22(tr?), IFAs: TMTD, phy |
| **SH50** | Shengavit | Trench K6, Locus 1127 | 73.9 | 101.6 | -28.7 | -27.9 | 0.9 | Non-ruminant adipose fats | SFA (C_14:0, 16:0, 18:0_), Alc (C_14, 18, 20_), Alk (C_20_), Hydroxy FAME (C_10, 11_), Sulfur |
| **SH63** | Shengavit | Trench K6, Locus 1107 | 76.8 | 206.1 | -25.8 | -30.1 | -4.3 | Dairy | SFA (C_14:0-25:0_), UFA (C_18:1_), Alc (C_18_), Alk (C_23_), Hydroxy FAME (C_24_), IFAs: phy |
| **SH68** | Shengavit | Trench K6, Locus 1167 | 548.5 | 1156.5 | -27.3 | -29.3 | -2.0 | Ruminant adipose fats and aquatic | SFA (C_14:0-16:0, 18:0-24:0, 26:0_), UFA (C_18:1_), Br (C_15, 17_), APAA C18, 20, 22?, IFAs: pris?, phy |
| **SH72** | Shengavit | Trench K6, Locus 1013 | 277.2 | 805.8 | -26.3 | -28.9 | -2.6 | Ruminant adipose fats | SFA (C_14:0-26:0, 28:0, 30:0_), UFA (C_18:1_), Br (C_17_), Alc (C_18_), Alk (C_19, 23_), IFAs: pris, phy |
| **SH82** | Shengavit | Trench M5, Locus 24028 | 110.9 | 176.4 | -26.5 | -30.1 | -3.6 | Dairy and plant(?) | SFA (C_14:0-18:0, 20:0, 24:0, 26:0_), UFA (C_18:1_), Alc (C_14, 17, 18, 20, 24, 26_), Alk (C_20, 22-29_), Hydroxy FAME (C_11, 15, 16_), Sulfur |
| **SH97** | Shengavit | Trench L4, Locus 7053 | 29.4 | 83.1 | -25.5 | -27.2 | -1.7 | Ruminant adipose fats and plant(?) | SFA (C_16:0-30:0_), Alc (C_16, 18_), Alk (C_17-33_) |
| **SH108** | Shengavit | Trench M5, Locus 24004 | 90.2 | 187.4 | -27.2 | -28.2 | -1.0 | Ruminant adipose fats and plant(?) | SFA (C_14:0-21:0, 24:0, 26:0, 28:0, 30:0_), Alc (C_16, 18, 19, 26, 28, 30_), Alk (C_17-25, 27, 29, 31, 33_) |
| **SH111** | Shengavit | Trench M5, Locus 24001 | 36.9 | 101.2 | -26.6 | -28.4 | -1.9 | Ruminant adipose fats and plant | SFA (C_16:0-18:0, 23:0, 24:0, 26:0, 28:0, 30:0_), Alc (C_14, 16, 18, 20, 22, 24, 26-28, 30_), Alk (C_19-31, 33_) |
| **SH112** | Shengavit | Trench M5, Locus 24001 | 31.3 | 79.0 | - | - | - | - | SFA (C_16:0, 18:0_), plasticizers |
| **MB001** | Mokhra-Blur | Levels (V-IX) | 102.9 | 296.5 | -24.8 | -27.3 | -2.4 | Ruminant adipose fats and plant(?) | SFA (C_14:0, 16:0-30:0, 32:0_), UFA (C_22:1, 18:1_), Alc (C_14, 26,, 28, 30_), Alk (C_17--27, 29_), Hydroxy FAME (C_24-26, 28_), Sulfur |
| **MB1** | Mokhra-Blur | Levels (V-IX) | 143.4 | 416.3 | -21.5 | -25.8 | -4.3 | Dairy and plant/resin? | SFA (C_14:0-24:0, 26:0-30:0, 32:0_), UFA (C_18:1_), Alc (C_18, 26, 28, 30_), Alk (C_21, 22_), Hydroxy FAME (C_24_), Sulfur, Dehydroabietic acid trimethylsilyl ester |
| **MB2** | Mokhra-Blur | Levels (V-IX) | 127.6 | 379.6 | -24.4 | -26.9 | -2.5 | Ruminant adipose fats | SFA (C_14:0, 16:0-18:0, 20:0-26:0, 28:0, 30:0_), UFA (C_18:1_), Alk (C_17-27, 29, 31, 33_), Hydroxy FAME (C_24_) |
| **MB3** | Mokhra-Blur | Levels (V-IX) | 2510.4 | 6593.3 | -22.4 | -25.5 | -3.1 | Dairy(?) and aquatic | SFA (C_14:0- 20:0, 22:0- 31:0_), UFA (C_18:1_), Br (C_15_), Alc (C_26_), Sulfur, APAA C18, 20(tr), 22(tr), IFAs: TMTD, pris, phy |
| **MB4** | Mokhra-Blur | Levels (V-IX) | 250.8 | 670.7 | -25.7 | -26.8 | -1.1 | Ruminant adipose fats | SFA (C_14:0-21:0_), Dehydroabietic trimethylsilyl ester, APAA C18? |
| **MB5** | Mokhra-Blur | Levels (V-IX) | 135.8 | 359.2 | -27.2 | -27.8 | -0.6 | Ruminant adipose fats | SFA (C_16:0, 18:0_), UFA (C_18:1_), Sulfur, Dehydroabietic acid, trimethylsilyl ester |
| **MB6** | Mokhra-Blur | Levels (V-IX) | 129.4 | 317.5 | -26.7 | -29.0 | -2.2 | Ruminant adipose fats and plant(?) | SFA (C_16:0, 18:0-30:0_), Alc (C_18, 24, 26, 28, 30_), Alk (C_20-33_), Sulfur, Dehydroabietic acid, trimethylsilyl ester |
| **MB7** | Mokhra-Blur | Levels (V-IX) | 32.3 | 94.2 | -25.8 | -27.8 | -1.9 | Ruminant adipose fats | SFA (C_14:0-18:0, 20:0, 24:0, 26:0, 28:0, 30:0_), UFA (C_18:1_), Alc (C_18, 24, 26, 28, 30_), Alk (C_27, 29, 31, 33_), Sulfur, Dehydroabietic acid, trimethylsilyl ester, APAA C18? |
| **MB8** | Mokhra-Blur | Levels (V-IX) | 85.9 | 251.3 | -25.6 | -26.2 | -0.6 | Ruminant adipose fats | SFA (C_14:0-18:0, 20:0_), UFA (C_18:1_), Alk (C_19--24_), Hydroxy FAME (C_12, 13, 15_), Dehydroabietic acid, trimethylsilyl ester, APAA C18 |
| **MB9** | Mokhra-Blur | Levels (V-IX) | 41.3 | 114.9 | -22.8 | -24.3 | -1.4 | Ruminant adipose fats and Plant/resin | SFA (C_14:0-20:0, 22:0, 24:0-29:0_), UFA (C_18:1_), Alk (C_21-32_), Dehydroabietic acid (TMS), 7-Oxodehydroabietic acid (ME) |
| **MB10** | Mokhra-Blur | Levels (V-IX) | 57.1 | 164.2 | -24.3 | -27.4 | -3.2 | Dairy | SFA (C_14:0, 16:0-18:0, 20:0, 22:0, 24:0, 28:0, 30:0_), Alc (C_18, 26, 28, 30_), Alk (C_19-22_), Hydroxy FAME (C_24, 26_), Sulfur, Dehydroabietic acid, trimethylsilyl ester, APAA C18 |
| **MB11** | Mokhra-Blur | Levels (V-IX) | 56.8 | 159.6 | -25.0 | -26.3 | -1.3 | Ruminant adipose fats and plant/resin | SFA (C_14:0, 16:0-18:0, 20:0, 22:0, 24:0, 26:0_), UFA (C_18:1, 22:1?_), Alc (C_14-16, 18, 24_), Alk (C_20-29_), Dehydroabietic acid (TMS), 7-Oxodehydroabietic acid (TMS) |
| **MB12** | Mokhra-Blur | Levels (V-IX) | 110.6 | 308.4 | -23.3 | -27.3 | -4.0 | Dairy | SFA (C_14:0-22:0, 24:0, 26:0, 28:0, 30:0, 32:0_), UFA (C_18:1_), Alc (C_18, 24, 26, 28, 30, 32_), Dehydroabietic acid (ME) |
| **KRT1** | Karnut-1 | Operation 1 | 100.5 | 282.4 | - | - | - | - | SFA (C_14:0-18:0, 20:0, 22:0-24:0_), UFA (C_16:1, 17:1, 18:1_), Hydroxy FAME (C_23, 24_) |
| **KRT2** | Karnut-1 | Operation 1 | 206.5 | 534.4 | - | - | - | Plant? | SFA (C_14:0-18:0, 20:0, 22:0-24:0_), UFA (C_16:1, 17:1?, 18:1_), Br (C_17_), Hydroxy FAME (C_23, 24_) |
| **KRT5** | Karnut-1 | Operation 1 | 202.9 | 479.5 | - | - | - | - | SFA (C_14:0-18:0, 20:0-24:0_), UFA (C_16:1, 17:1?, 18:1_), Br (C_17_) |
| **KRT6-6** | Karnut-1 | Operation 1 | 99.6 | 207.8 | - | - | - | - | SFA (C_14:0-24:0_), UFA (C_16:1, 18:1_), Br (C_17_) |
| **KRT7** | Karnut-1 | Operation 1 | 41.3 | 115.2 | -26.8 | -24.9 | 1.9 | Non-ruminant adipose fats | SFA (C_14:0-18:0, 20:0, 22:0, 23:0_), UFA (C_16:1, 18:1_), Br (C_17_) |
| **KRT8** | Karnut-1 | Operation 1 | 55.0 | 160.2 | - | - | - | - | SFA (C_14:0-18:0, 20:0, 22:0, 23:0, 24:0_), UFA (C_16:1, 17:1?, 18:1_), Br (C_17_) |
| **KRT12** | Karnut-1 | Operation 1 | 132.0 | 386.1 | -26.6 | -25.5 | 1.4 | Non-ruminant adipose fats and plant(?) | SFA (C_14:0-18:0, 22:0, 23:0_), UFA (C_16:1, 18:1_), Br (C_17_), Hydroxy FAME (C_23, 24_) |
| **KRT17** | Karnut-1 | Operation 1 | 65.8 | 187.2 | - | - | - | - | SFA (C_14:0-18:0, 22:0-24:0_), UFA (C_16:1, 18:1_), Alc (C_18_), Hydroxy FAME (C_24_) |
| **KRT20** | Karnut-1 | Operation 1 | 55.7 | 147.1 | -28.1 | -27.8 | 0.3 | Non-ruminant adipose fats? | SFA (C_14:0-18:0, 20:0, 22:0-24:0_), UFA (C_16:1, 17:1, 18:1_), Br (C_17_) |
| **KRT28** | Karnut-1 | Operation 1 | 83.9 | 218.1 | - | - | - | - | SFA (C_14:0-18:0, 20:0, 22:0-24:0_), UFA (C_16:1, 18:1_), Br (C_17_), Hydroxy FAME (C_24_) |
| **KRT33** | Karnut-1 | Operation 1 | 114.5 | 316.6 | - | - | - | - | SFA (C_14:0-18:0, 22:0-24:0_), UFA (C_16:1, 18:1_), Br (C_17_) |
| **KRT34** | Karnut-1 | Operation 1 | 70.8 | 204.0 | - | - | - | Plant? | SFA (C_14:0-18:0, 20:0, 22:0, 23:0_), UFA (C_16:1, 18:1_), Alc (C_18_), Hydroxy FAME (C_24_), APAA C18 |
| **KRT35** | Karnut-1 | Operation 1 | 171.8 | 490.8 | - | - | - | Plant wax | SFA (C_14:0-20:0, 22:0, 23:0_), UFA (C_16:1, 17:1, 18:1, 18:2_), Br (C_17_), Hydroxy FAME (C_23-25_), Terpenoids: Betulin (TMS) and unidentified terpenoids. |
| **KRT36** | Karnut-1 | Operation 1 | 21.1 | 59.9 | -27.7 | -29.1 | -1.4 | Ruminant adipose fats | SFA (C_14:0-18:0, 20:0, 23:0, 24:0_), UFA (C_18:1_), Alc (C_16, 18_), Hydroxy FAME (C_23, 24_) |
| **KRT41** | Karnut-1 | Operation 1 | 66.6 | 193.1 | -28.4 | -27.3 | 1.1 | Non-ruminant adipose fats | SFA (C_14:0-20:0, 22:0-24:0_), UFA (C_16:1, 17:1, 18:1_), Br (C_17_) |
| **KRT48** | Karnut-1 | Operation 1 | 79.0 | 230.1 | - | - | - | - | SFA (C_15:0-20:0, 22:0-24:0_), UFA (C_16:1, 17:1?, 18:1_), Br (C_17_) |
| **KRT52** | Karnut-1 | Operation 1 | 62.4 | 166.9 | - | - | - | - | SFA (C_15:0-24:0_), UFA (C_16:1, 18:1_), Br (C_17_) |
| **KRT53** | Karnut-1 | Trench 2, Room 1, Locus 3 (wall collapse) | 9.04 | 20.9 | -28.2 | -28.6 | -0.4 | Ruminant adipose fats | SFA (C_16:0, 18:0_), UFA (C_16:1, 18:1_) |
| **KRT55** | Karnut-1 | Trench 2, Room 1, Locus 3 (wall collapse) | 97.0 | 253.8 | -27.1 | -32.0 | -4.9 | Dairy | SFA (C_14:0-18:0_), UFA (C_18:1_), Alc (C_16, 18_), Dehydroabietic acid (TMS) |
| **KRT56** | Karnut-1 | Trench 2, Room 1, Locus 3 (wall collapse) | 297.0 | 813.3 | -27.7 | -33.1 | -5.4 | Dairy and plant(?) | SFA (C_14:0-26:0, 28:0, 30:0_), UFA (C_18:1, 18:2?_), Br (C_15, 17_), Alc (C_17, 18?_), Alk (C_29, 31, 33_), Dehydroabietic acid (TMS), APAA C18? IFAs: phy |
| **KRT57** | Karnut-1 | Trench 2, Room 1, Locus 3 (wall collapse) | 191.2 | 537.1 | -26.9 | -24.8 | 2.1 | Non-ruminant adipose fats | SFA (C_14:0-18:0_), UFA (C_18:1_), Alc (C_16, 18_), Dehydroabietic acid (TMS) |
| **KRT58** | Karnut-1 | Trench 2, Room 1, Locus 3 (wall collapse) | 35.2 | 104.6 | -27.7 | -27.7 | 0.0 | Non-ruminant adipose fats | SFA (C_16:0, 18:0_), UFA (C_18:1_), Alc (C_16, 18_), Dehydroabietic acid (TMS) |
| **KRT59** | Karnut-1 | Trench 2, Room 1, Locus 3 (wall collapse) | 55.9 | 158.6 | -26.6 | -30.0 | -3.4 | Dairy | SFA (C_14:0-18:0, 20:0, 22:0, 24:0-26:0_), UFA (C_18:1_), Alc (C_16, 18_), Dehydroabietic acid (TMS), APAA C18? |
| **KRT60** | Karnut-1 | Trench 2, Room 1, Locus 3 (wall collapse) | 85.3 | 235.5 | -27.8 | -32.4 | -4.6 | Dairy | SFA (C_14:0-16:0, 18:0_), UFA (C_18:1_), Dehydroabietic acid (TMS) |
| **KRT61** | Karnut-1 | Operation 2, B2 B3, Locus 2 | 77.4 | 218.0 | -27.9 | -29.6 | -1.7 | Ruminant adipose fats | SFA (C_16:0, 18:0_), UFA (C_18:1_) |
| **KRT62** | Karnut-1 | Operation 2, B2 B3, Locus 2 | 47.6 | 130.2 | -27.8 | -27.9 | -0.2 | Non-ruminant adipose fats? | SFA (C_14:0, 16:0-18:0_), UFA (C_18:1_), Alc (C_16, 18_), Dehydroabietic acid (TMS) |
| **KRT63** | Karnut-1 | Operation 2, B2 B3, Locus 2 | 78.4 | 185.3 | -27.1 | -26.4 | 0.6 | Non-ruminant adipose fats | SFA (C_16:0-18:0_), UFA (C_16:1, 18:1_), Dehydroabietic acid (TMS) |
| **KRT64** | Karnut-1 | Operation 2, B2 B3, Locus 2 | 126.4 | 372.2 | -28.4 | -30.0 | -1.6 | Ruminant adipose fats and plant(?) | SFA (C_14:0-16:0, 18:0, 20:0, 21:0, 24:0, 26:0, 28:0, 30:0_), UFA (C_18:1_), Alk (C_17-25, 27, 29, 31, 33_) |
| **KRT65** | Karnut-1 | Operation 2, B2 B3, Locus 2 | 34.3 | 90.6 | -27.5 | -26.8 | 0.7 | Non-ruminant adipose fats | SFA (C_16:0, 18:0_), UFA (C_18:1_), Alc (C_16, 18_), Hydroxy FAME (C_24_), Dehydroabietic acid (TMS) |
| **KRT66** | Karnut-1 | Operation 2, B2 B3, Locus 2 | 89.4 | 266.3 | -27.3 | -26.7 | 0.6 | Non-ruminant adipose fats | SFA (C_14:0-18:0_), UFA (C_18:1_) |
| **MG2** | Margahovit | M11-108, Trench A3 | 118.5 | 314.9 | -23.7 | -27.4 | -3.7 | Dairy and plant wax | SFA (C_14:0-26:0, 28:0_), UFA (C_16:1, 18:1_), Br (C_17_), Alc (C_14, 16, 18, 20-22, 24, 26, 28, 30_), Alk (C_23, 25-29, 31_), Diacid (C_16, 18, 20, 22_), $\omega$-Hydroxy FAME (C_22_), Hydroxy FAME (C_20, 24_), Terpenoids: Lup-2,20(29)-diene, Lup-2,20(29)-dien-28-ol (TMS), Allobetul-2-ene, Lupenone, Lupeol (TMS), unknown terpenoid, 28-oxoallobetul-2-ene?, Betulone (TMS), 3-oxoallobetulane, Betulin (TMS), Allobetulinol (TMS), IFAs: phy |
| **MG3** | Margahovit | M11-103, Trench A2 | 15.5 | 42.9 | -26.7 | -27.6 | -1.0 | Ruminant adipose fats and plant(?) | SFA (C_14:0-16:0, 18:0, 20:0, 24:0, 26:0, 28:0, 30:0_), UFA (C_16:1, 18:1_), Alc (C_14, 16, 18, 20, 22, 24, 26, 28_) |
| **MG5** | Margahovit | M11-85 | 8.49 | 18.0 | -27.4 | -28.0 | -0.6 | Ruminant adipose fats | SFA (C_16:0-18:0_), UFA (C_16:1, 18:1_), Alc (C_18_) |
| **MG7** | Margahovit | M13-1470 | 9.40 | 26.1 | - | - | - | - | - |
| **MG9** | Margahovit | M11-1679, Trench H6 | 431.5 | 1156.5 | -26.8 | -30.6 | -3.8 | Dairy and aquatic(?) | SFA (C_12:0-26:0, 28:0, 30:0_), UFA (C_16:1, 18:1_), Br (C_15, 17_), Alc (C_14, 18-20, 22, 24, 30_), IFAs: TMTD(tr), pris, phy |
| **MG10** | Margahovit | M13-1469 | 19.5 | 38.5 | -27.4 | -28.4 | -1.0 | Ruminant adipose fats | SFA (C_16:0, 18:0_), UFA (C_16:1, 18:1_), Alc (C_16, 18_) |
| **MG14** | Margahovit | M13-1342 | 15.9 | 36.0 | - | - | - | - | - |
| **MG15** | Margahovit | M14-1702, Trench H2 | 8.82 | 24.3 | -28.2 | -30.0 | -1.8 | Ruminant adipose fats | SFA (C_14:0-18:0_), UFA (C_16:1, 18:1_) |
| **MG16** | Margahovit | M11-286, Trench D3 | 14.5 | 30.1 | -26.4 | -27.9 | -1.5 | Ruminant adipose fats | SFA (C_15:0-18:0, 20:0, 24:0, 26:0_), UFA (C_16:1, 18:1, 22:1_), Alc (C_18, 20-22, 24_), Cholesterol, IFAs: pris? |
| **MG17** | Margahovit | M13-1090 | 41.2 | 9.54 | -27.3 | -31.2 | -3.8 | Dairy and aquatic | SFA (C_15:0-18:0, 20:0-26:0_), APAA C18, 20tr, 22tr, IFAs: phy(tr) |
| **MG19** | Margahovit | M14-1703, Trench H2 | 46.8 | 138.7 | -26.5 | -28.9 | -2.4 | Ruminant adipose fats and plant | SFA (C_15:0-18:0, 20:0-30:0_), Alc (C_20, 22, 24, 26, 28_), Alk (C_23-31, 33_) |
| **MG20** | Margahovit | M14-1573, Trench H6 | 6.61 | 17.5 | -27.6 | -28.9 | -1.3 | Ruminant adipose fats and plant? | SFA (C_16:0-18:0, 20:0, 24:0, 26:0_), UFA (C_16:1, 18:1, 22:1_) |
| **MG21** | Margahovit | M13-271 | 41.7 | 117.0 | - | - | - | - | SFA (C_14:0-18:0, 20:0, 22:0, 24:0, 26:0, 28:0, 30:0_), UFA (C_18:1, 20:1_), Br (C_17_), Alc (C_14, 18, 20, 22, 24, 26_), Cholesterol |
| **MG22** | Margahovit | M12-711 | 2.39 | 6.85 | - | - | - | - | - |
| **MG23** | Margahovit | M11-300, Trench C3 | 1.97 | 5.61 | - | - | - | - | SFA (C_15:0-18:0_), UFA (C_16:1, 18:1_), IFAs: TMTD?, pris? |
| **MG24** | Margahovit | M12-708 | 5.81 | 15.5 | -28.0 | -29.4 | -1.4 | Ruminant adipose fats | SFA (C_16:0-18:0_), UFA (C_18:1_) |
| **MG26** | Margahovit | M13-915, Trench B5 | 15.3 | 40.4 | -27.4 | -29.6 | -2.3 | Ruminant adipose fats and plant(?) | SFA (C_16:0-18:0, 20:0, 24:0, 26:0_), UFA (C_18:1_), Alc (C_18, 20-22, 24, 26_), Sulfur, Cholesterol, IFAs: TMTD?, pris? |
| **MG27** | Margahovit | M13-1179, Trench G8 | 19.8 | 55.5 | -27.1 | -28.7 | -1.7 | Ruminant adipose fats | SFA (C_16:0-18:0, 20:0, 22:0, 24:0, 26:0, 28:0, 30:0_), UFA (C_18:1_), Alc (C_16, 18, 24, 26, 28, 30_), Alk (C_29, 31, 33_) |
| **MG28** | Margahovit | M11-62 | 49.9 | 139.4 | -27.1 | -31.9 | -4.8 | Dairy | SFA (C_14:0-26:0, 28:0, 30:0_), UFA (C_16:1, 18:1_), Br (C_17_), Alc (C_19_), Hydroxy FAME (C_16, 24_), IFAs: phy |
| **MG29** | Margahovit | M14-917, Trench B5 | 29.2 | 84.3 | -27.3 | -31.4 | -4.2 | Dairy and aquatic(?) | SFA (C_14:0-26:0, 28:0, 30:0_), UFA (C_18:1_), Alc (C_18, 24, 26_), IFAs: TMTD (tr?), pris (tr?), phy |
| **MG30** | Margahovit | M11-244, Trench D4 | 22.6 | 61.3 | -27.6 | -30.7 | -3.1 | Dairy and plant(?) | SFA (C_16:0, 18:0, 20:0_), UFA (C_16:1, 18:1_), Alc (C_16-18, 20_), Cholesterol? |
| **MG31** | Margahovit | M13-967, Trench C2 | 18.6 | 50.0 | - | - | - | - | SFA (C_16:0-20:0, 26:0, 28:0, 30:0_), UFA (C_16:1, 18:1_), Alc (C_14, 16, 18-20, 26, 28, 30_) |
| **MG33** | Margahovit | M13-1384, Trench F5 | 57.4 | 122.4 | -26.1 | -28.3 | -2.2 | Ruminant adipose fats and aquatic | SFA (C_14:0-24:0, 26:0, 30:0_), UFA (C_16:1, 18:1_), Br (C_17_), Alc (C_16, 18, 20-22, 24, 26, 30_), Diacid (C_16_?), IFAs: TMTD, pris, phy |
| **MG34** | Margahovit | M11-263, Trench D2a | 31.3 | 67.0 | -27.2 | -28.8 | -1.6 | Ruminant adipose fats | SFA (C_16:0-18:0_), UFA (C_16:1, 18:1, 18:2?, 20:1_), Alc (C_16-18_) |
| **MG39** | Margahovit | M14-1711, Trench H2 | 7.54 | 20.0 | -27.3 | -27.9 | -0.6 | Ruminant adipose fats | SFA (C_16:0, 18:0_), UFA (C_18:1_) |
| **MG40** | Margahovit | M12-525 | 4.57 | 10.0 | - | - | - | - | SFA (C_16:0, 18:0_), UFA (C_18:1_) |
| **MG43** | Margahovit | M11-65, Trench A4 | 276.3 | 813.3 | -26.9 | -31.2 | -4.3 | Dairy and plant(?) | SFA (C_13:0-30:0, 32:0_), UFA (C_18:1_), Br (C_17_), Alc (C_12?, 16, 18, 20, 22, 24, 26, 28, 30, 32_), Alk (C_19, 21, 23-33_), Hydroxy FAME (C_15_), Ketones (C_31, 33?_), IFAs: pris, phy |
| **MG44** | Margahovit | M13-957, Trench C5 | 12.6 | 33.5 | -26.3 | -26.5 | -0.2 | Non-ruminant adipose fats | SFA (C_15:0-20:0, 23:0, 24:0, 28:0_), UFA (C_16:1, 18:1_), Alc (C_16, 18, 20, 26_), Hydroxy FAME (C_24_), |
| **MG45** | Margahovit | M12-735, Trench F1 | 10.0 | 22.8 | - | - | - | - | SFA (C_16:0, 18:0_), UFA (C_18:1_), Alc (C_16, 18, 20_) |
| **MG46** | Margahovit | M13-1265 | 7.53 | 16.3 | -26.7 | -27.3 | -0.6 | Ruminant adipose fats | SFA (C_16:0-18:0_), UFA (C_16:1, 18:1_), Alc (C_18_) |
| **T1** | Talin Tombs | Tomb ‘cult’ structure number 10 & 12 | 87.1 | 251.3 | -26.1 | -29.7 | -3.6 | Dairy | SFA (C_14:0-18:0, 24:0_), UFA (C_18:1_), Alc (C_12, 14-18_), Alk (C_19-24_), Hydroxy FAME (C_24_) |
| **T2** | Talin Tombs | Tomb ‘cult’ structure number 10 & 12 | 359.7 | 1001.2 | -27.0 | -31.1 | -4.2 | Dairy | SFA (C_14:0-26:0_), UFA (C_18:1_), Br (C_17_), Alc (C_14, 16, 18-20_), Alk (C_22_), IFAs: phy |
| **T5** | Talin Tombs | Tomb ‘cult’ structure number 10 & 12 | 408.2 | 1111.8 | -26.3 | -28.2 | -1.9 | Ruminant adipose fats | SFA (C_14:0-30:0_), Alc (C_16, 18, 26, 28, 30_), Alk (C_22-26_) |
| **T7** | Talin Tombs | Tomb ‘cult’ structure number 10 & 12 | 193.3 | 141.8 | -26.8 | -31.2 | -4.4 | Dairy | SFA (C_14:0-17:0, 18:0, 20:0, 22:0, 24:0_), UFA (C_18:1_), Alc (C_14, 16_), Alk (C_17, 19-24_), Hydroxy FAME (C_11_) |
| **SK6** | Sotk-2 | S11-345, Trench C, Unit 1 | 138.6 | 389.1 | -27.0 | -29.7 | -2.7 | Ruminant adipose fats and plant(?) | SFA (C_12:0-18:0, 20:0_), UFA (C_16:1, 17:1, 18:1, 22:1_), Alc (C_12, 16-24_), Alk (C_22, 24-30, 31?, 33?_), Cholesterol, IFAs: TMTD?, pris? |
| **SK23** | Sotk-2 | S11-95, Trench C, Unit 3 | 27.4 | 64.3 | -22.2 | -25.6 | -3.4 | Dairy and plant(?) | SFA (C_14:0-18:0, 20:0-28:0_), UFA (C_16:1, 18:1_), Alc (C_16-18, 26_), Alk (C_24, 26-31_), IFAs: TMTD, pris? |
| **SK46** | Sotk-2 | S12-747, Trench F, Unit 2 | 158.2 | 445.1 | -27.6 | -29.7 | -2.0 | Ruminant adipose fats | SFA (C_14:0-24:0_), UFA (C_18:1_), Br (C_17_), IFAs: phy |
| **SK51** | Sotk-2 | S12-803, Trench F, Unit 2 | 143.3 | 350.4 | -27.1 | -32.6 | -5.5 | Dairy | SFA (C_14:0-26:0_), UFA (C_16:1, 18:1_), Br (C_17_), Alc (C_14-16, 18_), Alk (C_18-33_), IFAs: phy |

**References for Supplementary Table (S2)**

Correa-Ascencio M, Evershed RP. High throughput screening of organic residues in archaeological potsherds using direct methanol extraction. Analytical Methods. 2014;6: 1330-1340.
