# Supplementary material for: Diverse dietary practices across the Early Bronze Age ‘Kura-Araxes culture’ in the South Caucasus
Source: PLoS One. 2022 Dec 21;17(12):e0278345. doi: 10.1371/journal.pone.0278345 (PMC9770345; doi:10.1371/journal.pone.0278345)
Supplement: S3 Table — P/S ratios, CPI, Paq, and classification of trimethylsilylated TLEs. P/S ratio = relative abundance ratio of C16:0/C18:0 fatty acids, where values greater than 4 indicate a plant origin. CPI = measures the relative abundance of odd-over-even carbon chain lengths; CPI values for all plant species have strong odd-chain preferences, ranging between 1.6 and 82.1 [99, 100, 127]. Paq = emergent and non-emergent aquatic macrophyte input; Paq <0.1 corresponds to a terrestrial plant input; Paq 0.1–0.4 to emergent macrophytes, and Paq 0.4–1.0 to submerged or floating macrophytes [101]. N/D–not determined, signal intensity too low. (DOCX) [file pone.0278345.s007.docx]

# **S3 Table. Plant lipid calculations.**

P/S ratios, CPI, *P*_aq_ , and classification of trimethylsilylated TLEs. P/S ratio = relative abundance ratio of C_16:0_/C_18:0_ fatty acids, where values greater than 4 indicate a plant origin. CPI = measures the relative abundance of odd-over-even carbon chain lengths; CPI values for all plant species have strong odd-chain preferences, ranging between 1.6 and 82.1 (Diefendorf et al., 2011; Bush and McInerney, 2013; Herrera-Herrera et al., 2020). *P*_aq_ = emergent and non-emergent aquatic macrophyte input; *P*_aq_ <0.1 corresponds to a terrestrial plant input; *P*_aq_ 0.1-0.4 to emergent macrophytes, and *P*_aq_ 0.4-1.0 to submerged or floating macrophytes (Ficken et al., 2000). N/D – not determined, signal intensity too low.

**Equations**

P/S ratio: relative abundance ratio of C_16:0_/C_18:0_ fatty acids, values greater than 4 indicate a possible plant origin.

$$CPI= \frac{1}{2} \left( \frac{(C_{25}+C_{27}+C_{29}+C_{31}+C_{33})}{(C_{24}+C_{26}+C_{28}+C_{30}+C_{32})} +\frac{(C_{25}+C_{27}+C_{29}+C_{31}+C_{33})}{(C_{26}+C_{28}+C_{30}+C_{32}+C_{34})} \right)$$

$$P_{aq}= (C_{23}+ C_{25})/(C_{23}+ C_{25}+ C_{29}+ C_{31})$$

| **Laboratory Code** | **Site** | ***P*_aq_** | **CPI** | **P/S** | **Classification** |
| --- | --- | --- | --- | --- | --- |
| SH21 | Shengavit | 0.48 | 2.7 | 11.3 | Plant |
| SH32 | Shengavit | 0.23 | 18.8 | 0.5 | Plant |
| SH45 | Shengavit | N/D | N/D | 5.6 | Possible Plant |
| SH79 | Shengavit | 0.57 | 2.9 | 9.7 | Plant |
| SH82 | Shengavit | 0.85 | 0.9 | 1.2 | Possible Plant |
| SH97 | Shengavit | 0.19 | 3.3 | 2.4 | Plant |
| SH105 | Shengavit | 0.52 | 1.6 | 11.2 | Possible Plant |
| SH108 | Shengavit | 0.55 | N/D | 0.2 | Possible Plant |
| SH111 | Shengavit | 0.31 | 2.3 | 0.3 | Plant |
| SH132 | Shengavit | N/D | N/D | 0.1 | Plant wax |
| MB001 | Mokhra-Blur | 1.00 | 2.0 | 3.8 | Possible Plant |
| MB1 | Mokhra-Blur | N/D | N/D | 1.2 | Possible Plant resin |
| MB6 | Mokhra-Blur | 0.22 | 5.0 | 1.0 | Plant |
| MB9 | Mokhra-Blur | 0.84 | 1.1 | 0.7 | Plant resin |
| MB11 | Mokhra-Blur | 0.78 | N/D | N/D | Plant resin |
| MG2 | Margahovit | 0.00 | 69.0 | 1.1 | Plant wax |
| MG3 | Margahovit | N/D | N/D | 1.6 | Possible Plant |
| MG19 | Margahovit | 0.14 | 16.5 | 0.6 | Plant |
| MG20 | Margahovit | N/D | N/D | 2.1 | Possible Plant |
| MG26 | Margahovit | N/D | N/D | 1.7 | Possible Plant |
| MG30 | Margahovit | N/D | N/D | 2.5 | Possible Plant |
| MG31 | Margahovit | N/D | N/D | 3.6 | Possible Plant |
| MG43 | Margahovit | 0.14 | 1.5 | 0.4 | Possible Plant |
| MG46 | Margahovit | N/D | N/D | 3.5 | Possible Plant |
| SK6 | Sotk-2 | 0.39 | 0.8 | 3.2 | Possible Plant |
| SK23 | Sotk-2 | 0.00 | 1.05 | 1.2 | Possible Plant |
| KRT2 | Karnut-1 | N/D | N/D | 7.8 | Possible Plant |
| KRT12 | Karnut-1 | N/D | N/D | 4.9 | Possible Plant |
| KRT34 | Karnut-1 | N/D | N/D | 7.2 | Possible Plant |
| KRT35 | Karnut-1 | N/D | N/D | N/D | Plant wax |
| KRT56 | Karnut-1 | N/D | N/D | 1.0 | Possible Plant |
| KRT64 | Karnut-1 | 0.81 | N/D | 1.1 | Possible Plant |
| G28 | Gegharot | N/D | N/D | 0.9 | Plant wax |
| G31 | Gegharot | N/D | N/D | 0.8 | Plant wax |

**References for Supplementary Table (S3)**

Bush RT, McInerney FA. Leaf wax *n*-alkane distributions in and across modern plants: implications for paleoecology and chemotaxonomy. Geochimica et Cosmochimica Acta. 2013;117, 161-179.

Diefendorf AF, Freeman KH, Wing SL, Graham HV. Production of *n*-alkyl lipids in living plants and implications for the geologic past. Geochim. Cosmochim. Acta. 2011;75, 7472-7485.

Ficken KJ, Li B, Swain DL, Eglinton G. An *n*-alkane proxy for the sedimentary input of submerged/floating freshwater aquatic macrophytes. Organic Geochemistry. 2000;31, 745-749.

Herrera-Herrera AV, Leierer L, Jambrina-Enríquez M, Connolly R, Mallol C. Evaluating different methods for calculating the Carbon Preference Index (CPI): Implications for palaeoecological and archaeological research. Organic Geochemistry. 2020;146: 104056. <https://doi.org/10.1016/j.orggeochem.2020.104056>.
